# Supplementary material for: Lithium intercalation into bilayer graphene
Source: Nat Commun. 2019 Jan 17;10:275. doi: 10.1038/s41467-018-07942-z (PMC6336798; doi:10.1038/s41467-018-07942-z)
Supplement: Supplementary file 1 — Supplementary Information [file 41467_2018_7942_MOESM1_ESM.pdf]

Supplementary information

Lithium intercalation into bilayer graphene

Ji *et al.*

Supplementary Figures

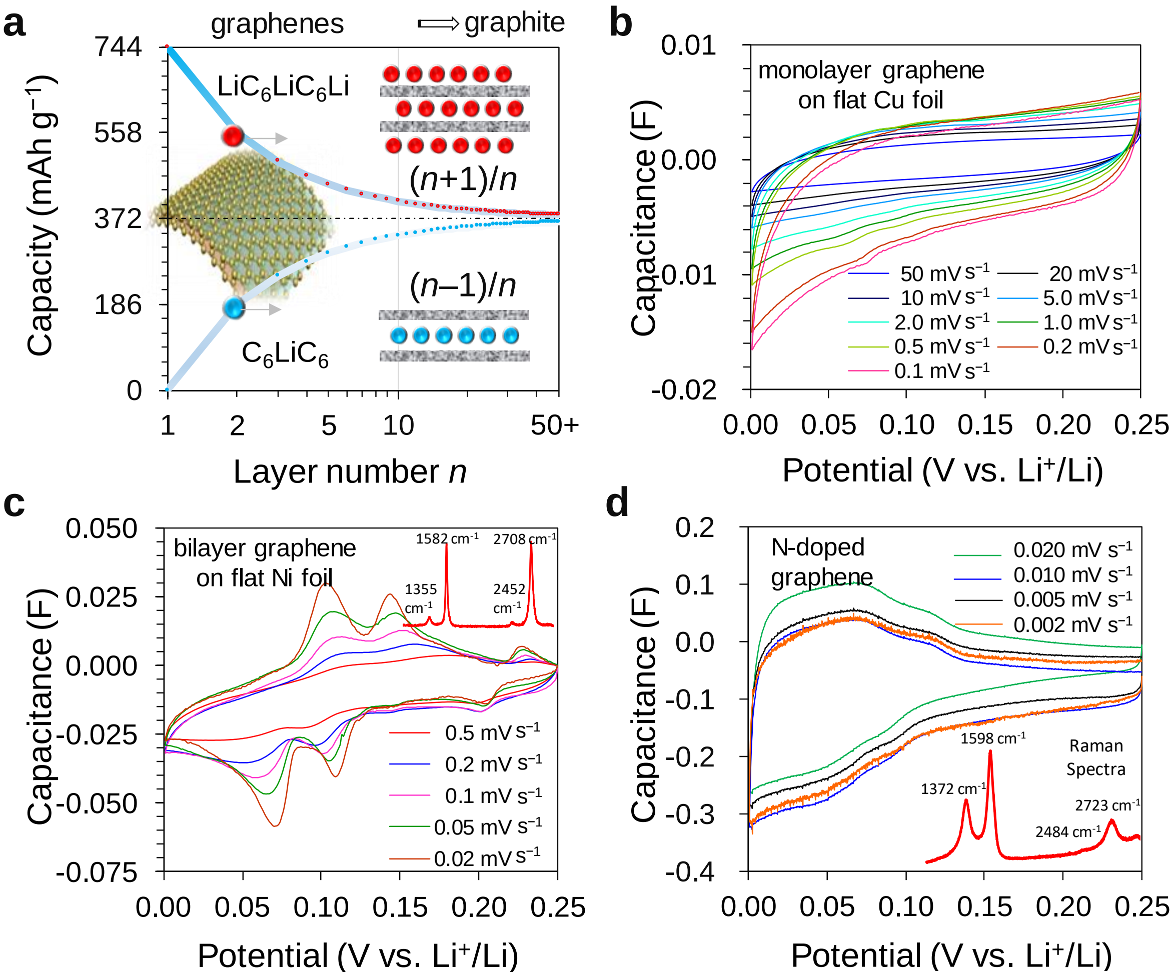

Supplementary Fig. 1 | Model-dependent capacities and measured CVs of various graphenic carbons. a, Theoretical Li-storage capacities of graphenic carbons dependent on the layer numbers (monolayer (n = 1), fewlayer (n = 2–5), and multilayer (n = 6–10))[1] based on the two viewpoints mentioned in the text. The insets display the deductive configurations of lithiated bilayer graphene under the two conditions, where the orthohexagnal C_6 ring serves as the minimum storage unit for each Li atom to form the theoretical and empirical LiC_6 stoichiometry of 372 mAh g^{-1}. **b**, CVs of monolayer graphene on flat Cu foil at the range of 0.25–0.001 V. It should be the space between the graphene sheet and Cu substrate to contribute such capacitive-like behavior[2–4]. **c**, CVs of bilayer graphene on flat Ni foil at 0.25–0.001 V. The Ni substrate would also induce capacitive behavior to the graphene/Ni composite, in particular at relatively high sweep rates (e.g., 0.5 mV s^{-1}). Only when the sweep rate is small enough (e.g., 0.02 mV s^{-1}), there would emerge the featured CV redox peaks for graphenes/graphite electrodes. **d**, Capacitive CVs of N atom-doped multilayer graphene foam at 0.25–0.001 V, illustrating the impact of rich defects on the Li-storage behavior. The insets in (**c**) and (**d**) showed the typical Raman spectra of the applied graphenic carbons.

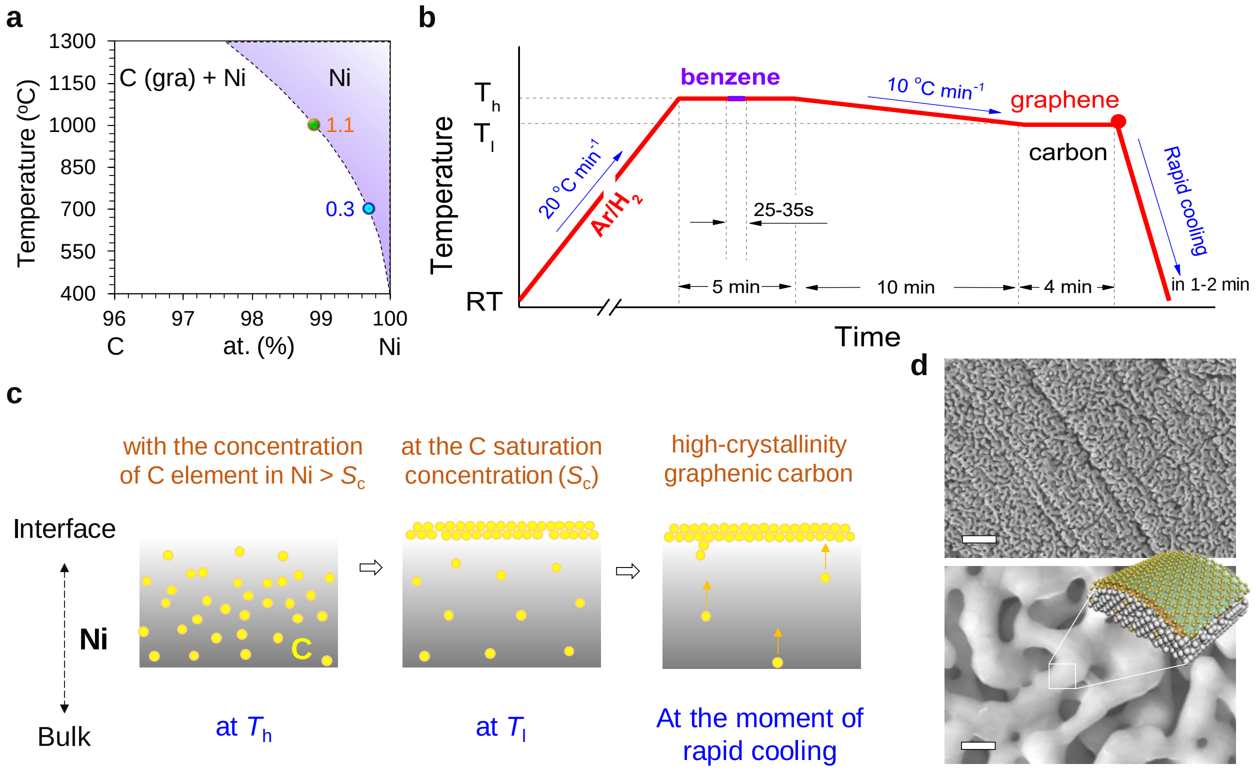

Supplementary Fig. 2 | Preparation of bilayer graphene foam. a, C-Ni phase diagram[5]. **b**,**c**, CVD program to prepare bilayer graphene at a high-temperature range using 3D nanoporous Ni substrate (*np*-Ni, 0.8 cm × 1.2 cm × 30 μm) and the possible growth mechanism[6,7]. This fabrication generally followed an ever-proposed process in *ref.* 8. However, in considering the C-Ni phase diagram (**a**) and the growth mechanism of graphene on Ni (i.e., the cooling-induced segregation of the dissolved C atoms)[9], the previous CVD process was divided into three stages as shown in (**b**) and Fig. 1a, such as the C-swallow period at a high temperature T_h (at 850−950 °C, lasting for 5 min including the carbon-dissolution time), the C-spitting period at a low temperature T_l (at 750−850 °C, lasting for 4 min), and the in-between slow temperature-fall period (ΔT = 100 °C, 10 °C min^{-1}) for the uniform C diffusion in the bulk of Ni. On the one hand, such an operation can efficiently limit the carbon uptake and release to enable bilayer graphenic carbon to generate on Ni at T_l[6] rather than at the moment of rapid cooling as before[8,10] (**b**, **c**). On the other hand, due to the resistance from the improving graphene crystal with less defects and the decreased diffusivity of C element in the fast-cooling Ni (in 15−20 s from the displayed T_l to 400 °C and then ca. 1 min to 60 °C, **b**), it would also restrain the precipitation of excess C atoms at the Ni-graphene interface to yield high-quality bilayer graphene[7,10] (**c**). **d**, SEM images of the as-prepared graphene@*np*-Ni composite (scale bar, 5 μm and 500 nm, respectively).

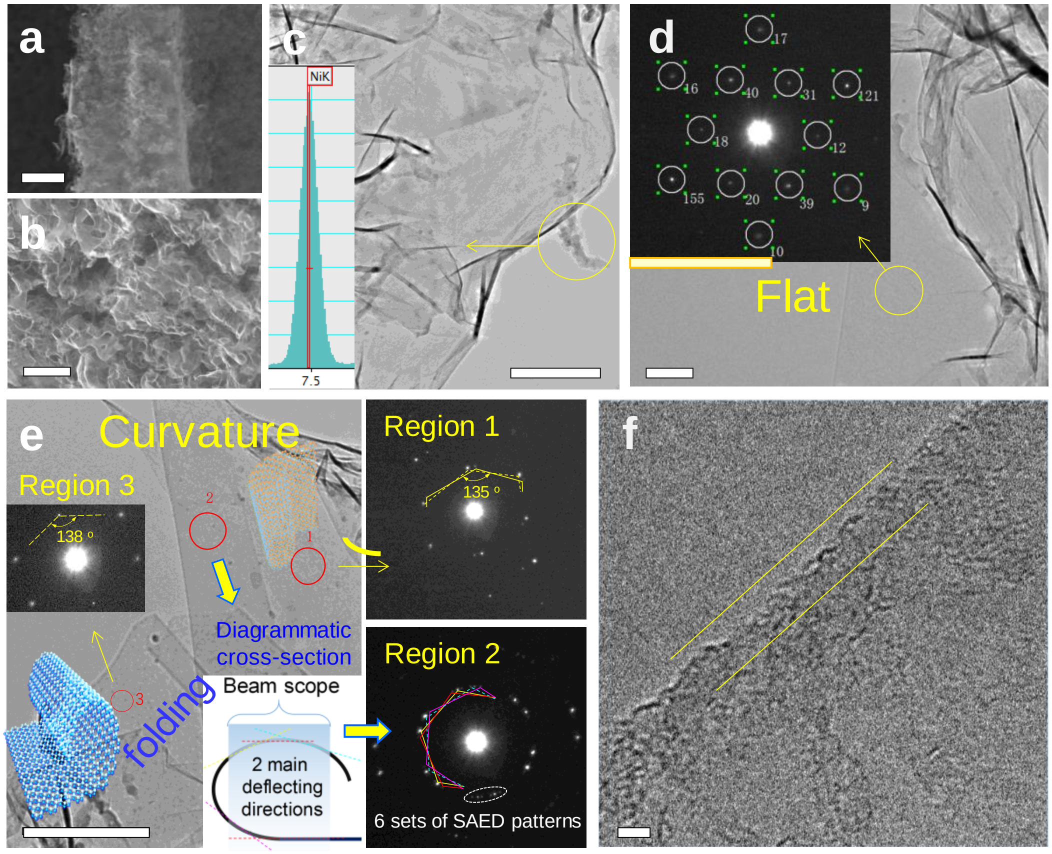

Supplementary Fig. 3 | Microstructure identification of the bilayer-graphene foam. a,**b**, SEM images of freestanding bilayer-graphene foam (~30 μm thick) exfoliated from 3D porous Ni foam. **c**−**e**, Typical TEM images and SAED patterns from different regions of the bilayer-graphene sample. The (**c**) inset showed the residual Ni. The lattice spacings for the {0110} and {1210} planes were measured to be ca. 2.12 and 1.23 Å from the normal-incidence SAED pattern in (**d**, namely Fig. 1d; scale bar, Å^{-1}), respectively, approximate to the theoretical 2.13 and 1.23 Å. The $I_{\{0110\}}/I_{\{1210\}}$ intensity was estimated according to the measured brightness of each diffraction peak (namely the numbers marked in the pattern), as follows: $I_{\{0110\}}/I_{\{1210\}} = (40 + 31 + 12 + 39 + 20 + 18)/(17 + 121 + 9 + 10 + 155 + 16) \approx 0.488$. The bended/curly graphene sheets (i.e., Regions 1 and 3 in (**e**)) exhibited distorted hexagonal diffraction patterns, quite different from the orthohexagnal one from the flat part (**d**) and indicating the effects of curvature[8,11,12], when the local fold area (e.g., Region 2 in (**e**) covering two layers of pristine graphene sheets) gave more than two sets of diffraction patterns[13,14]. Quite local curvature, corrugation, or misorientation inevitable for a suspended but flat graphene sheet seem not to change the $I_{\{0110\}}/I_{\{1210\}}$ ratio too much in view of the previously reported values stable at 0.4−0.5 for various bilayer graphenes[11,15,16] and our simulated 0.44 (Fig. 3d). **f**, Typical HR-TEM image collected from the sheet edge. Due to the weak diffraction intensity and grain contrast as well as the tilt angle between the electron beam and the normal direction to the selected graphene sheet, the as-obtained TEM images tended to show barely differentiable lattice and disturbed crystal planes[11,17,18]. The scale bars refer to 10 μm, 2 μm, 200 nm, 50 nm, 200 nm, and 2 nm in (**a**−**f**), respectively.

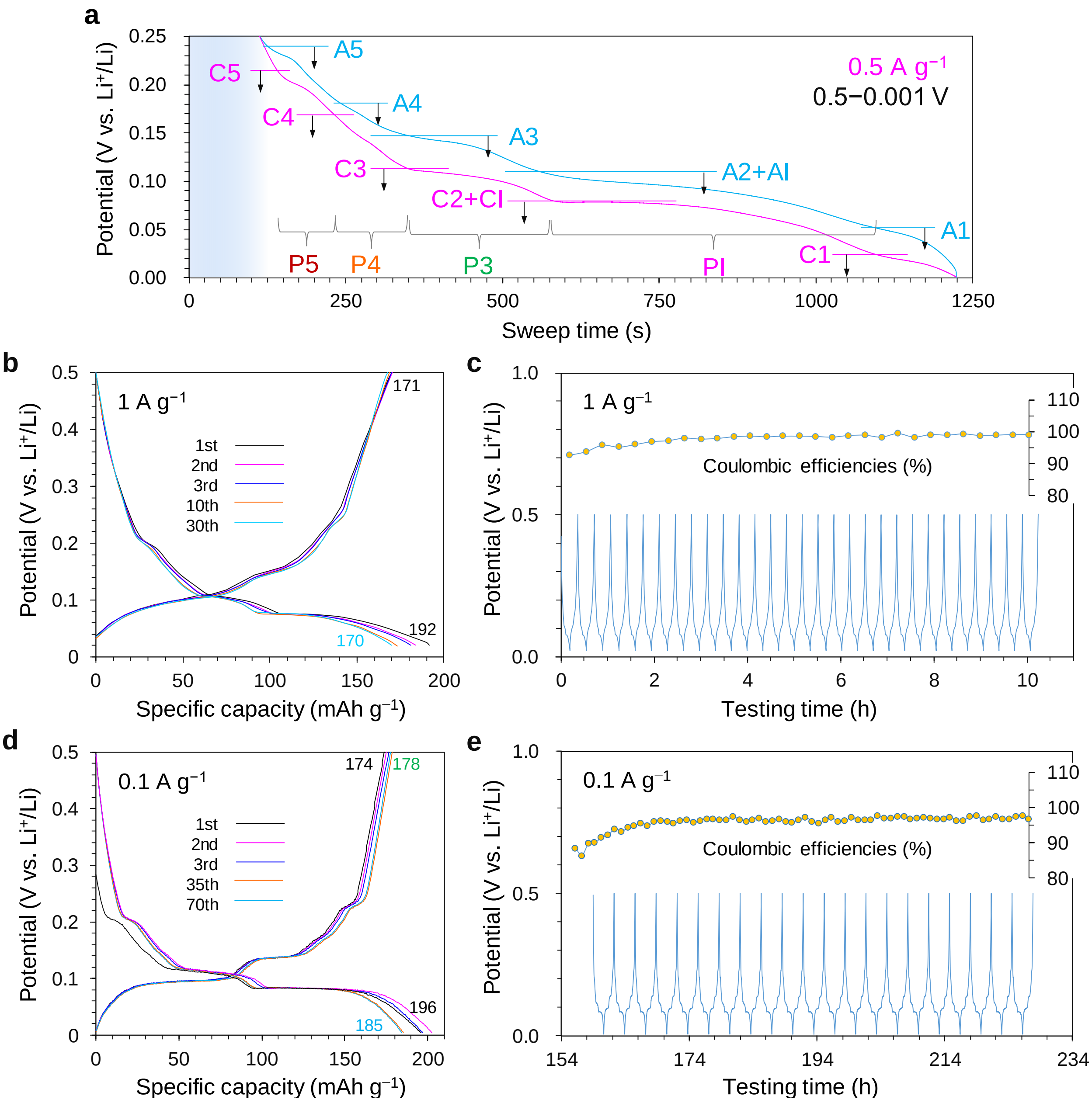

Supplementary Fig. 4 | CD performances the bilayer-graphene foam electrode. a, CD curves the bilayer-graphene foam electrode at 0.5 A g^{-1} between 0.5 and 0.001 V (reproduced from the inset of Fig. 2a, refer to *ref.* 19), consistent with the CV observations (Fig. 2b). **b**,**c**, CD profiles (1−30 circles) of one used bilayer-graphene foam electrode (after the CV test) at 1.0 A g^{-1} between 0.5 and 0.02 V. **d**,**e**, CD profiles (1−70 circles) of one fresh bilayer-graphene electrode at 0.1 A g^{-1} between 0.5 and 0.005 V. These results verified both the stable enough performance of the 3D nanoporous bilayer-graphene material during a long period of testing and the exemption of the serious influence from the surface SEI (generated at > 0.5 V during the initial electrochemical cycles), guaranteeing the reliability of our experimental results.

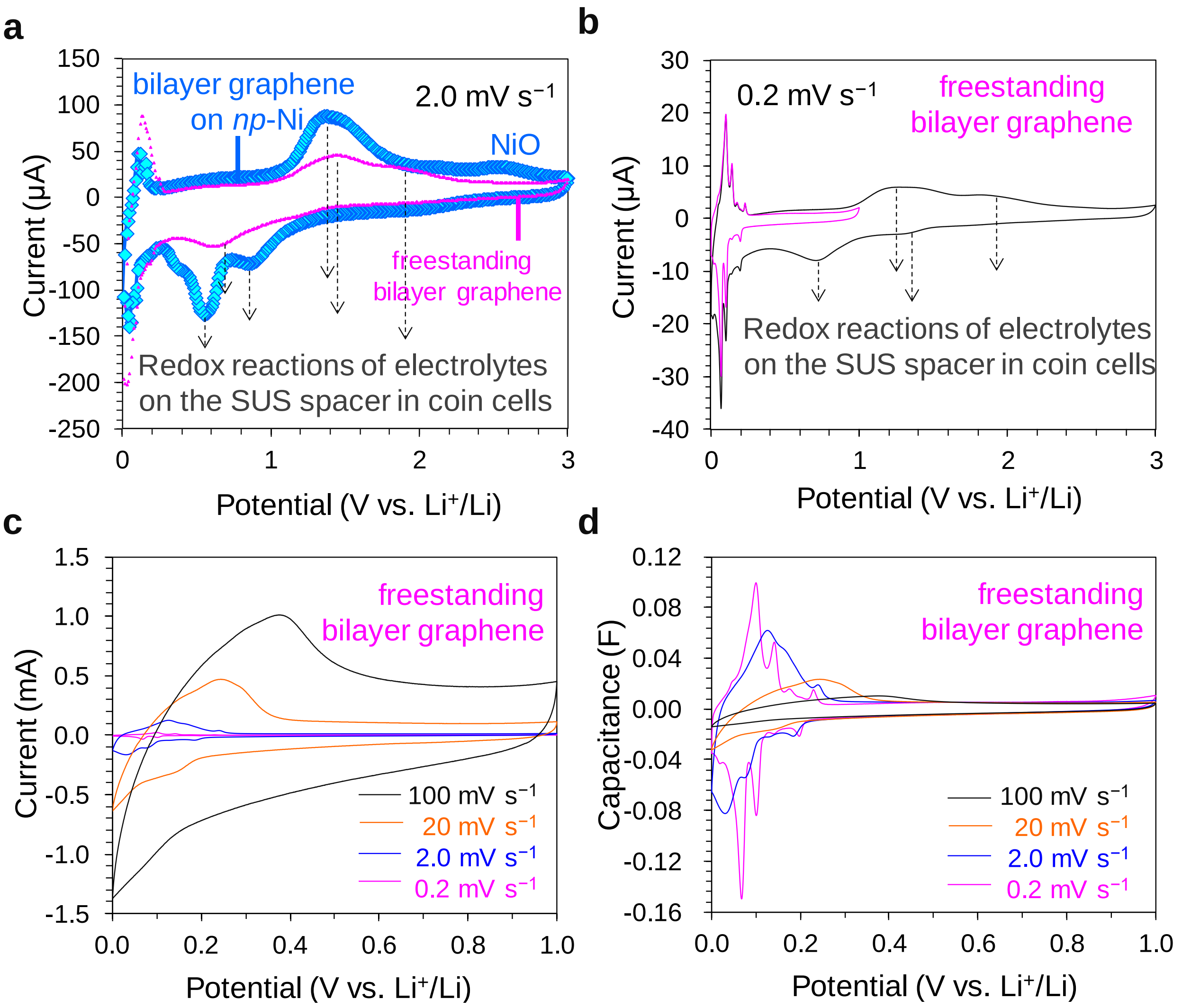

Supplementary Fig. 5 | Evolution of the CVs of the bilayer-graphene foam electrode. a, Comparative CVs of the bilayer graphene@*np*-Ni composite and the freestanding bilayer-graphene foam at 2.0 mV s^{-1}, indicating the influence of Ni substrate. **b**, CVs of the freestanding bilayer-graphene foam at 0.2 mV s^{-1} at varied potential ranges. The attribution of the redox peaks above 0.5 V can refer to *refs* 3 and 4. No NiO or stainless steel (SUS) spacer-related peaks emerged after the upper limited voltage decreased to 1 V, illustrating the exemption from some other side reactions of our measurements. **c**,**d**, Different forms for CVs of freestanding bilayer graphene foam at 100 to 0.2 mV s^{-1}, which clearly display the evolutive CV peak shapes and an ever-present CV hysteresis loop (see its related discussion at Supplementary Fig. 11c).

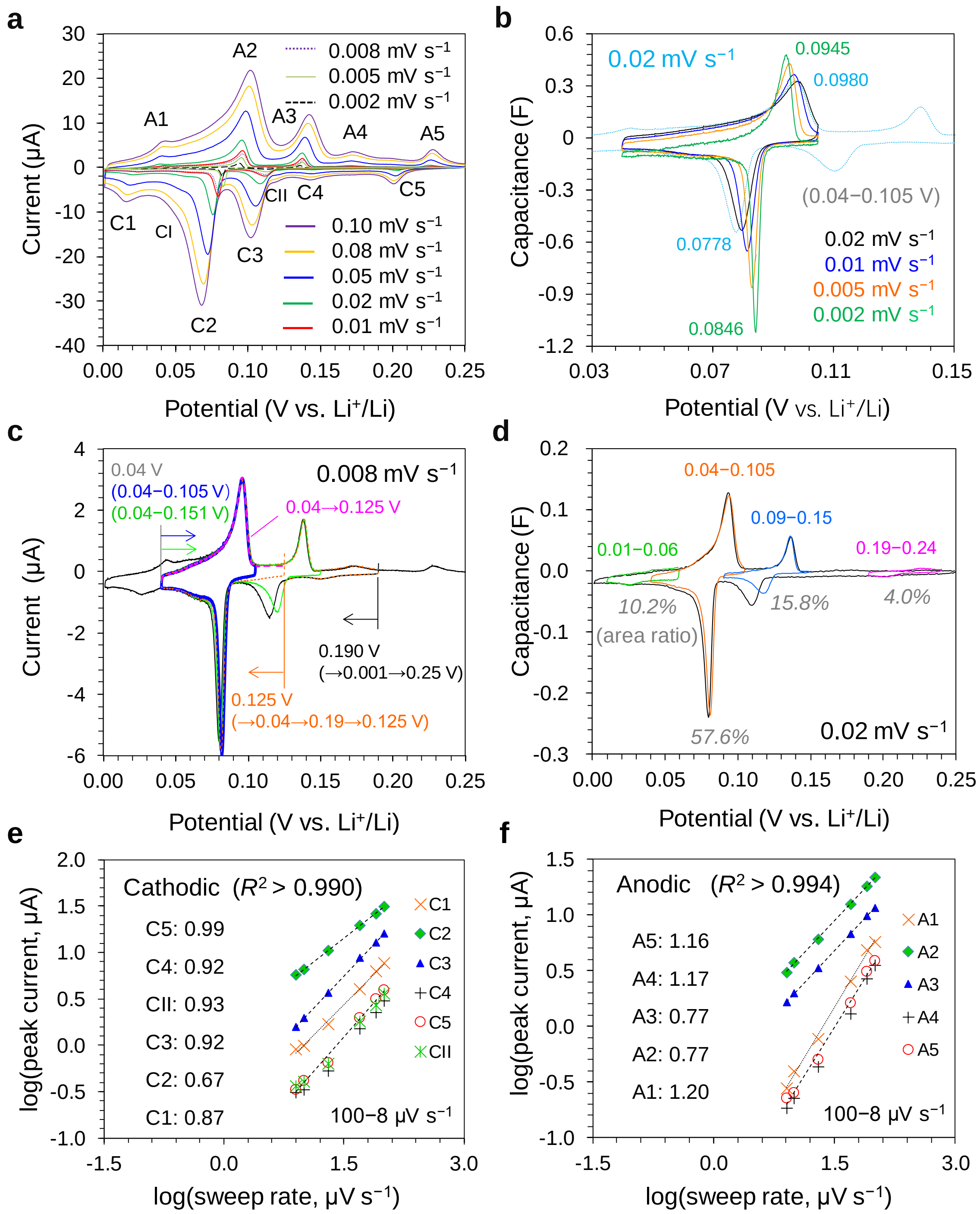

Supplementary Fig. 6 | Identification of the CV features of the bilayer-graphene foam electrode. a, CVs varied by 0.10 to 0.002 mV s^{-1} at the range of 0.25–0.001 V (vs. Li^+/Li) for the freestanding bilayer-graphene foam. **b**, CVs from 0.02 to 0.002 mV s^{-1} at 0.105–0.04 V (vs. Li^+/Li), which exhibit the corresponding relation of C_2 and A_2 peaks as well as the intrinsic existence of the CI shoulder peak. **c**,**d**, CVs performed at alternative potential ranges at 0.008 mV s^{-1} (**c**) and 0.02 mV s^{-1} (**d**), respectively. **e**,**f**, Kinetics analysis of the *b* values based on the cathodic and anodic peak currents displayed in (**a**).

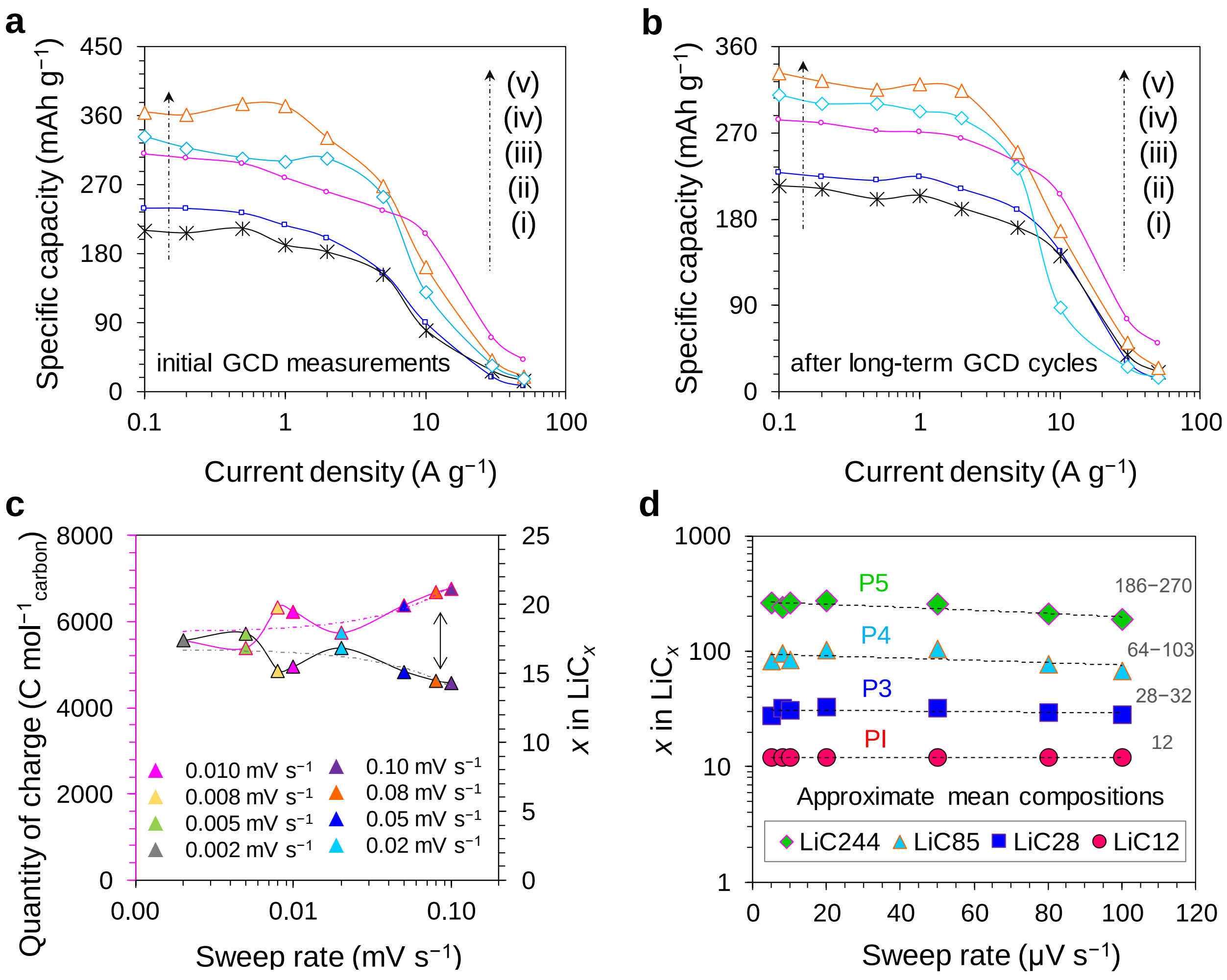

Supplementary Fig. 7 | Identification of the energy storage performances of the bilayer-graphene foam electrode. a,**b**, Specific capacities for 3D nanoporous graphene samples prepared at varied experimental conditions (e.g., time and temperature in Supplementary Fig. 2b) as a function of CD current densities ranged from 50 to 0.1 A g^{-1}. Thereinto, for the (i) and (ii) samples, the T_h and T_l values were 850 and 750 °C, respectively, which were 950 and 850 °C for the (iii) and (v) samples, when the (v) sample was prepared following the procedure described in *ref.* 20. Their performances were measured both at the initial period (**a**) and after the GCD tests at 0.2 A g^{-1} for 30−50 circles (**b**), respectively. The results show that the graphene samples' capacities were always lower than 372 mAh g^{-1} of graphite, and suggest that the average graphene layers were 2−3, ~3, and 4−6 for the (i−iii) samples and > 8 for the (vi) and (v) samples (refer to Supplementary Fig. 1a), in line with their Raman features (refer to Fig. 1e). **c**, Specific quantity of electric charge of the bilayer-graphene sample (theoretically at 8040 C mol^{-1} for LiC_{12}) and the corresponding average number of C atoms in the stoichiometric LiC_x units based on the CV performances in Supplementary Fig. 6a. **d**, Average compositions of various phases by calculating individual peak areas over the CV curves in Supplementary Fig. 6a.

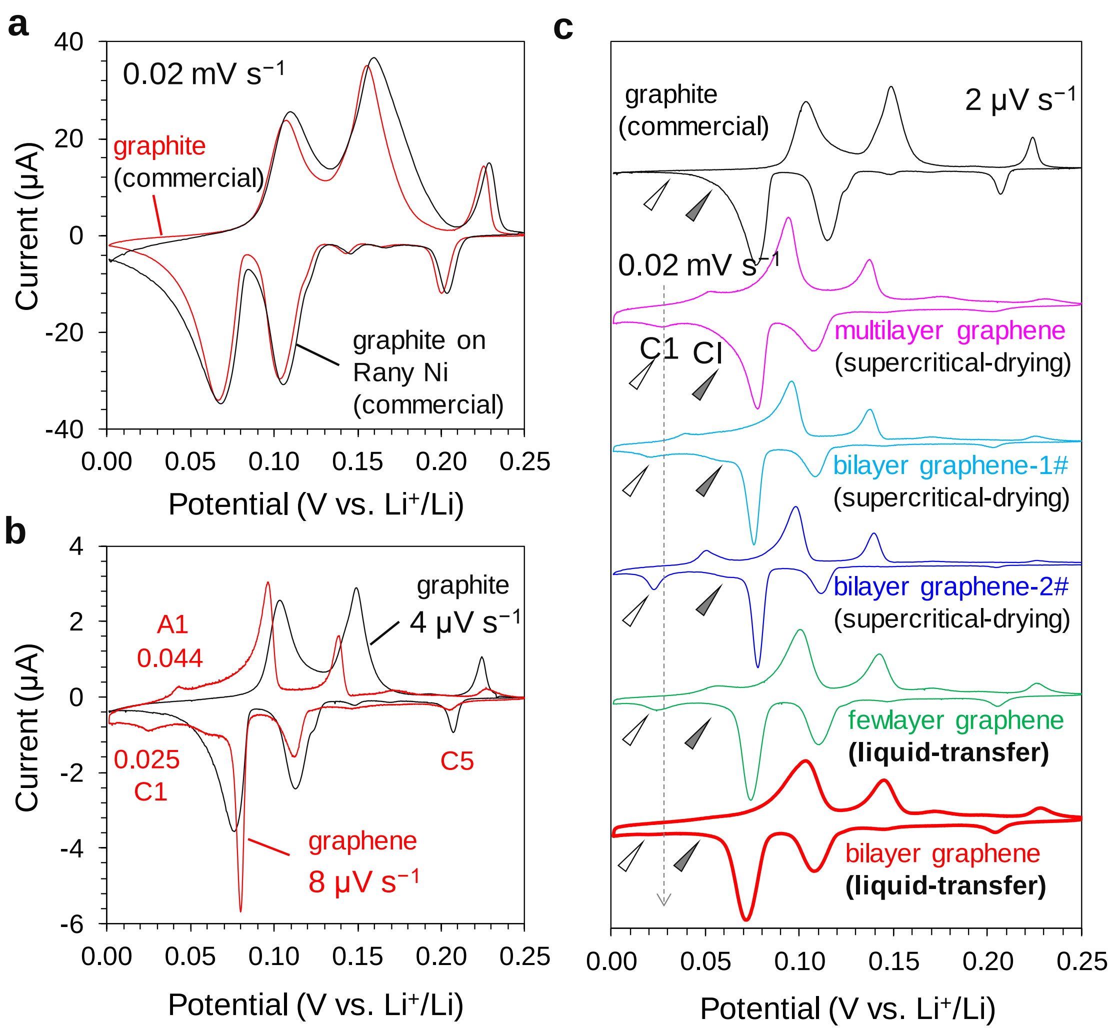

Supplementary Fig. 8 | Comparative CVs of various graphenic carbons. a, CV profiles at 0.02 mV s^{-1} of the commercial graphite electrodes supported by Rany Ni substrate or not. **b**, Comparative CV profiles at 4 μV s^{-1} of the freestanding bilayer-graphene foam and commercial graphite. **c**, Comparative CV profiles of the graphene foams and graphite electrodes obtained by different post-processing routes. The bilayer graphene 1# (950−850 °C) and 2# (850−750 °C) samples with different defective ratios were both obtained through the supercritical-drying process. The liquid-transferred sample means that the as-prepared graphene foam was directly assembled into the coin cell after removing Ni in HCl aqueous and being washed in the EC-DMC solution (without the drying process). The C1 peak (as well as the C5 peak) was noticed to vary a lot in different samples, when the CV shapes at the CI position (marked by grey triangles) were similar for the multilayer graphene sample and graphite electrode. It is worth mentioning that, graphite electrodes would also exhibit changeable CV shapes due to their specific preparations (and thus defects)[21].

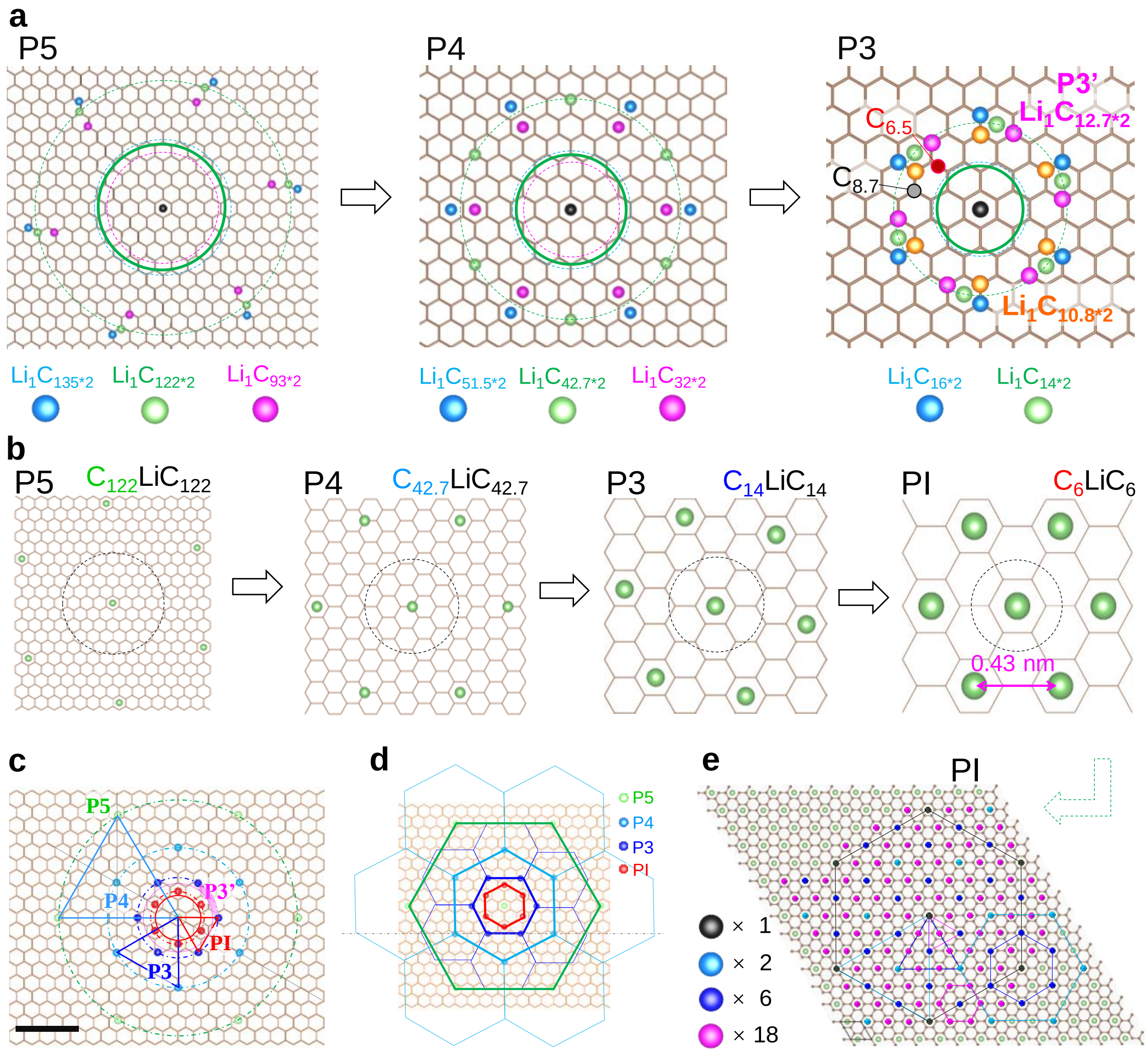

Supplementary Fig. 9 | Comparative analyses for the planar images of the possible LiC_x compositions. a, Ideally areal distribution of Li atoms on the X-Y plane according to the estimated Li-intercalation/deintercalation process shown in Supplementary Fig. 7d, namely the range and the mean of the seven x values for each LiC_x phase. It can be seen that every Li atom can locate at the C_6-ring center or vertex, which from geometry view is also necessary to keep the large-range periodicity and ordering of the microstructure corresponding to each LiC_x unit (refer to the regular XPS results in Fig. 4). According to this rule, the as-claimed Li-graphite intercalation product of LiC_{12}, if this estimation was relatively accurate, may be revised as $LiC_{12.7}$ (P3') compared with the other adjacent viable composition of $LiC_{10.8}$, as shown in the P3 image. By the way, the succedent $C_{8.7\text{–}6.5}LiC_{8.7\text{–}6.5}$ next to C_6LiC_6 is perhaps close to the stoichiometric composition at the transitional P2 phase (with an unstable quasi-SP stacking configuration between A*R*B and A*R*A as discussed at Supplementary Fig. 16). However, judging from the highly overlapping CV peaks

of strong C2 and weak CI (Fig. 2b and Supplementary Figs 6 and 8) and the following XRD (Supplementary Fig. 16) and XPS results (Fig. 4 and Supplementary Fig. 21), the real LiC_x product at this transient state seem to become the mixture of the PI and P3 (or more) intercalation compounds[22]. **b**, Distribution of Li atoms on the graphene network according to the average stoichiometric LiC_x phases, namely C ⇋ LiC_{122} ⇋ $LiC_{42.7}$ ⇋ LiC_{14} ⇋ LiC_6. **c**,**d**, Geometrical position relationships of Li atoms at the staged Li-graphene phases based on the data in (**b**). The scale bar in (**c**) refers to 1 nm. The inset three equilateral triangles in (**c**) indicate that the incoming Li atoms (except for the PI formation marked by the solid red circle) can happen to locate at the geometric centers of three adjacent Li atoms in the present phase. Each vertex of the inset color-coded regular hexagons in (**d**) refers to one Li atom generated at the corresponding phases. After each Li intercalation, there will emerge 6 Li atoms around each Li atom in the former phase until achieving the Li-saturated state in the graphene interlayer, yielding a "visual expression" of fractal-way Li intercalation. **e**, Theoretical planar image of LiC_6 plotted following the reverse fractal Li-intercalation way, in which the color-coded balls refer to the intercalated Li atoms at each step. As a result, the "backward" series from C_6LiC_6 is $C_6LiC_6 \rightarrow C_{18}LiC_{18} \rightarrow C_{54}LiC_{54} \rightarrow C_{162}LiC_{162} \rightarrow$ C. In this hypothetic mode, the three main intermediate phases (P5, P4, and P3) possess much lower Li distribution densities than the above experimental ones, which would not increase the Li^+ diffusion resistances greatly at the 2D space even for the final formation of C_6LiC_6 because of the always large enough distances between any two neighbouring Li atoms.

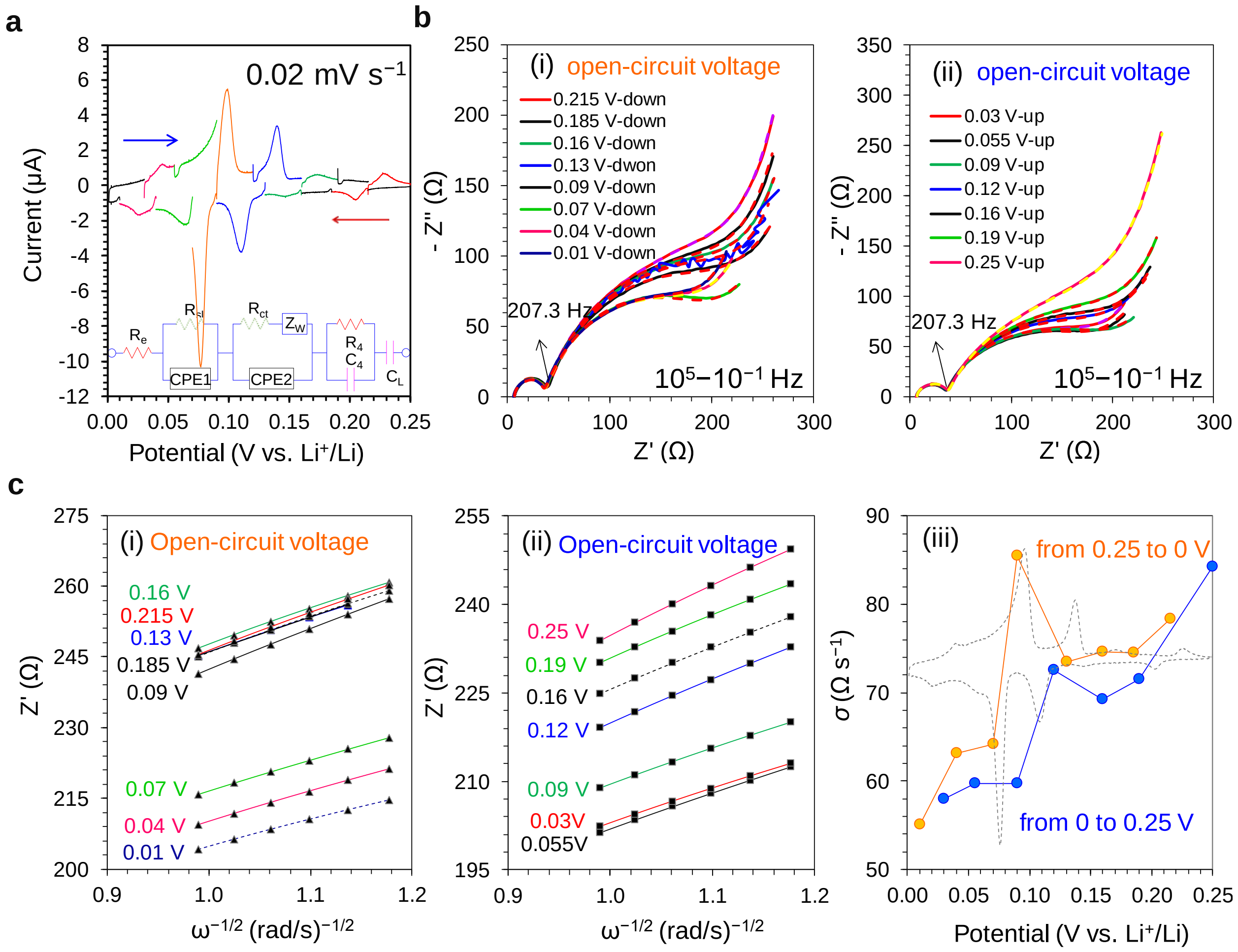

Supplementary Fig. 10 | EIS measurement and Li-diffusion analysis for the bilayer-graphene foam electrode. a, Intermittent CVs at 0.02 mV s^{-1} to obtain every phase for the Nyquist plots in (**b**). Specifically, when reaching a preset voltage, the workstation stopped automatically and the cut-off voltage was set as the open-circuit voltage for the prompt EIS measurement. The (**a**) inset (amplified in Supplementary Fig. 11a) refers to the ever-proposed equivalent circuit to simulate the Nyquist curves of graphite electrode[23]. **b**, Experimental (the solid lines) and simulative (the dashed lines) EIS curves. The rugged plot at 0.13 V in (i) should relate to the aberrant CV behavior for the P3 formation (Supplementary Fig. 6c,d). **c**, Plots of the real part of impedance (Z') as a function of the inverse square root of angular frequency ($\omega^{-1/2}$) in the Warburg region (i.e., the frequency ranges of the slope with 45° in the Nyquist curves). From the slopes of Z' versus $\omega^{-1/2}$ ($Z_W = \sigma\,(1\text{−j})\,\omega^{-1/2}$) (i, ii), the values of Warburg coefficients σ at each open-circuit voltage were calculated firstly, as plotted in (iii). Thus, apparent chemical diffusion coefficients of Li^+ (i.e., D_{Li^+}) (Fig. 2e) could be further obtained according to the equation of $\sigma = RT/\left((2D_{Li^+})^{1/2} n^2 F^2 A_{\Delta}C_{Li}\right)$[23–27]. Thereinto, (1) R is the gas constant; (2) T is the room temperature; (3) n is the number of electron taking part in the intercalation reaction; (4) F is the Faraday constant (96486 C mol^{-1}); (5) A is the area of electrode (ca. 0.05 cm^2 and 0.026 mg for the as-applied sample here); and (6) $_{\Delta}C_{Li}$ is the changed concentration of Li in the lithiated graphene material.

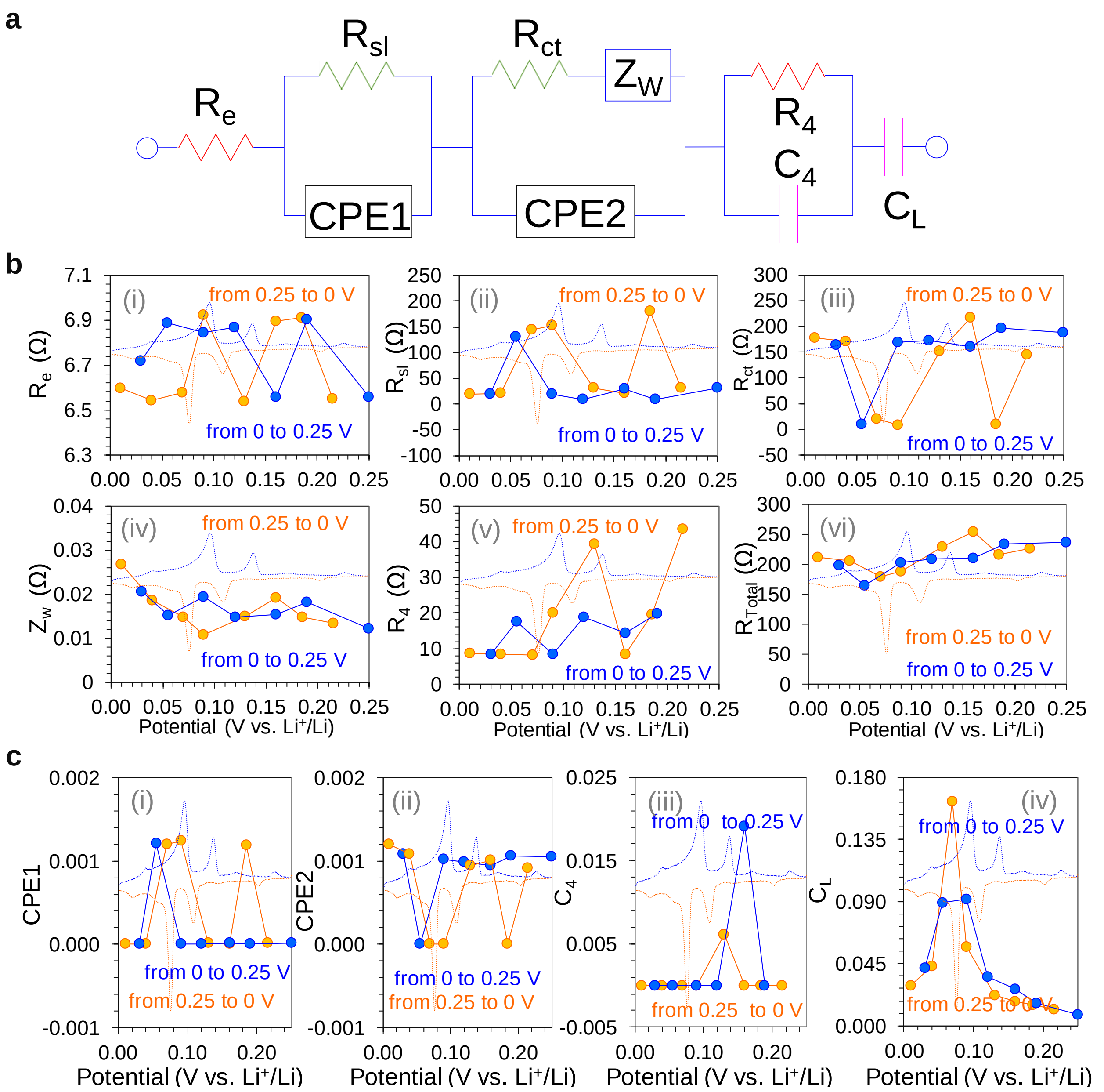

Supplementary Fig. 11 | Simulation analysis of the AC impedances for the bilayer-graphene foam electrode. a, Equivalent circuit for the simulation of the Nyquist plots in Supplementary Fig. 10b. Thereinto, (1) R_e is the total resistance of the electrode, electrolyte, and separator; (2) R_{sl} is the resistance of the SEI film, and CPE1 refers to its space charge capacitance; (3) R_{ct} is the charge transfer resistance at the electrode-SEI interface, CPE2 refers to the double-layer capacitance based on the surface roughness of the electrode, and Z_w is the Warburg impedance owing to Li diffusion in the electrode; (4) the $C_4||R_4$ unit is inserted into the circuit to subsume the contribution of the SEI-solution interface, where C_L is regarded as the insertion capacitance around the measured potential[23]. The resulting chi-squared functions were ~10^{-3} and the largest errors for the resulting parameters were less than 10%[23]. **b**, Evolution of various charge transfer resistances, such as (i) R_e, (ii) R_{sl}, (iii) R_{ct}, (iv) Z_w, (v) R_4, and (vi) the total resistance R_{Total}. It can be observed that

the SEI film led to the vast majority of the system resistance[23] (~90% for R_{ct} + R_{sl}), for which the graphene electrode itself only contributed < 3%. Associated with the Li^+ diffusion in the bilayer-graphene interlayer, the Warburg impedance Z_w always achieved the maximum value at P1 in either the Li-intercalation or its inverse processes. Besides, R_{Total} decreased by some extent along with the Li intercalation[23]. **c**, Evolution of the capacitive-like units. The changes of CPE1 and CPE2 ($\Omega^{-1}\cdot s^n$, in which a n-value of ~0.5 refers to a semi-infinite diffusion controlled electrochemical behaviour, and a n-value of ~1.0 represents a capacitor-like surface controlled electrochemical behaviour) kept pace with their parallel resistances R_{sl} and R_{ct}, respectively. Their values varied alternatively between exact 0 (n = 0.98−1.0) and ca. 0.0012 (n = 0.75−0.80), suggesting the influence of the stacking configuration or Li concentration (refer to the changed spatial relationship of local Li and C atoms in Supplementary Fig. 9b and Scheme 1b) on the SEI interface. C_L became much larger at the A*R*A stacking C_6LiC_6 phase and the ever-present CV hysteresis loop (Supplementary Fig. 5d) may be associated with this parameter.

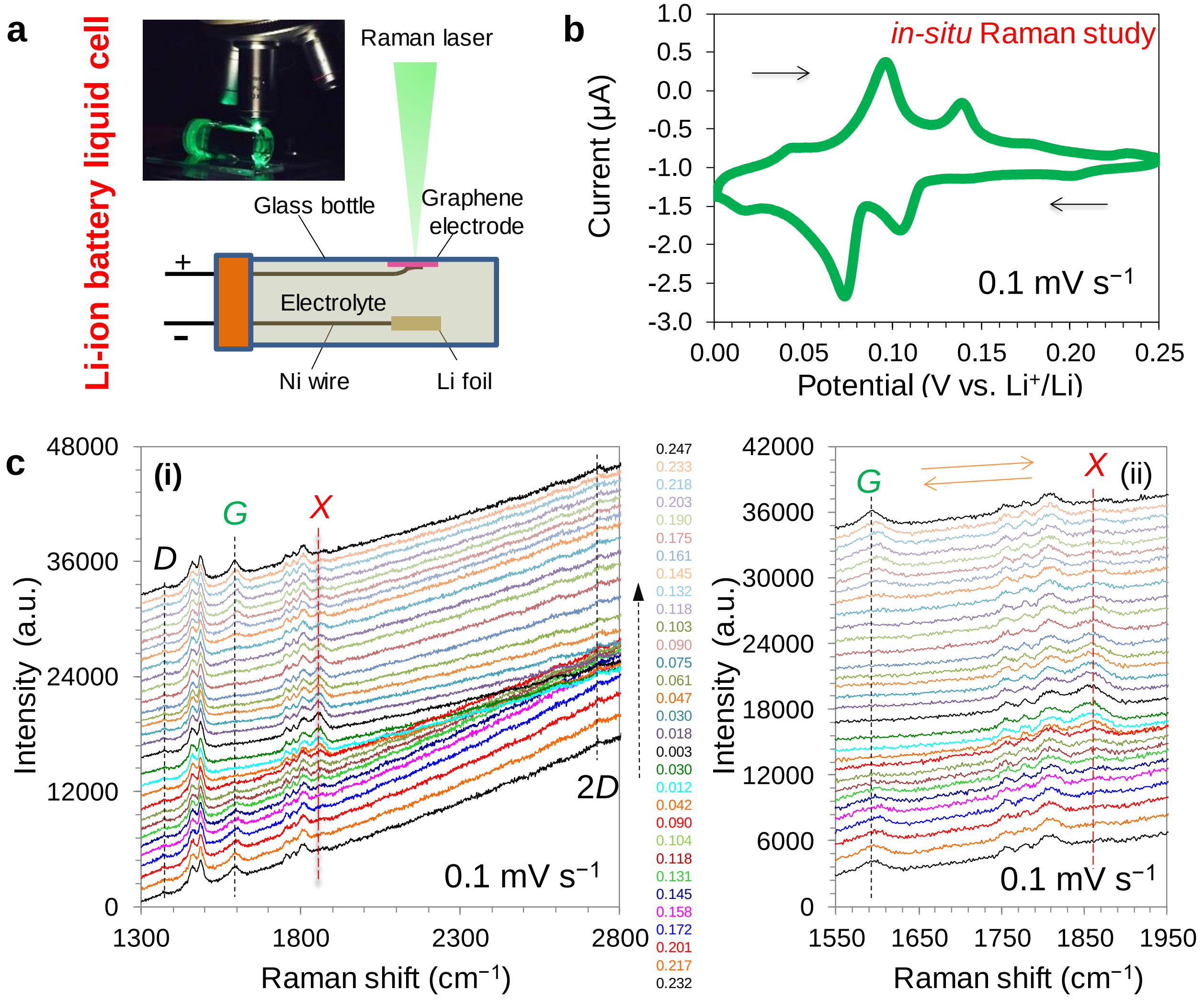

Supplementary Fig. 12 | *In-situ* Raman spectra measurements for the graphene foam. a, Schematic of the liquid-cell device for the *in-situ* Raman study. **b**,**c**, CV curve at 0.1 mV s^{-1} (**b**) for the *in-situ* Raman spectra shown in (**c**), for which the CV voltage ran from 0.25 to 0.001 V firstly and then returned to 0.25 V. The *G* peak tended to vanish after 0.090 V during the Li intercalation process, and to reappear after 0.103 V during the Li deintercalation process. The intensity evolution of the emerging *X* band assigned to the electrolyte showed its close relationship with the intercalated Li population.

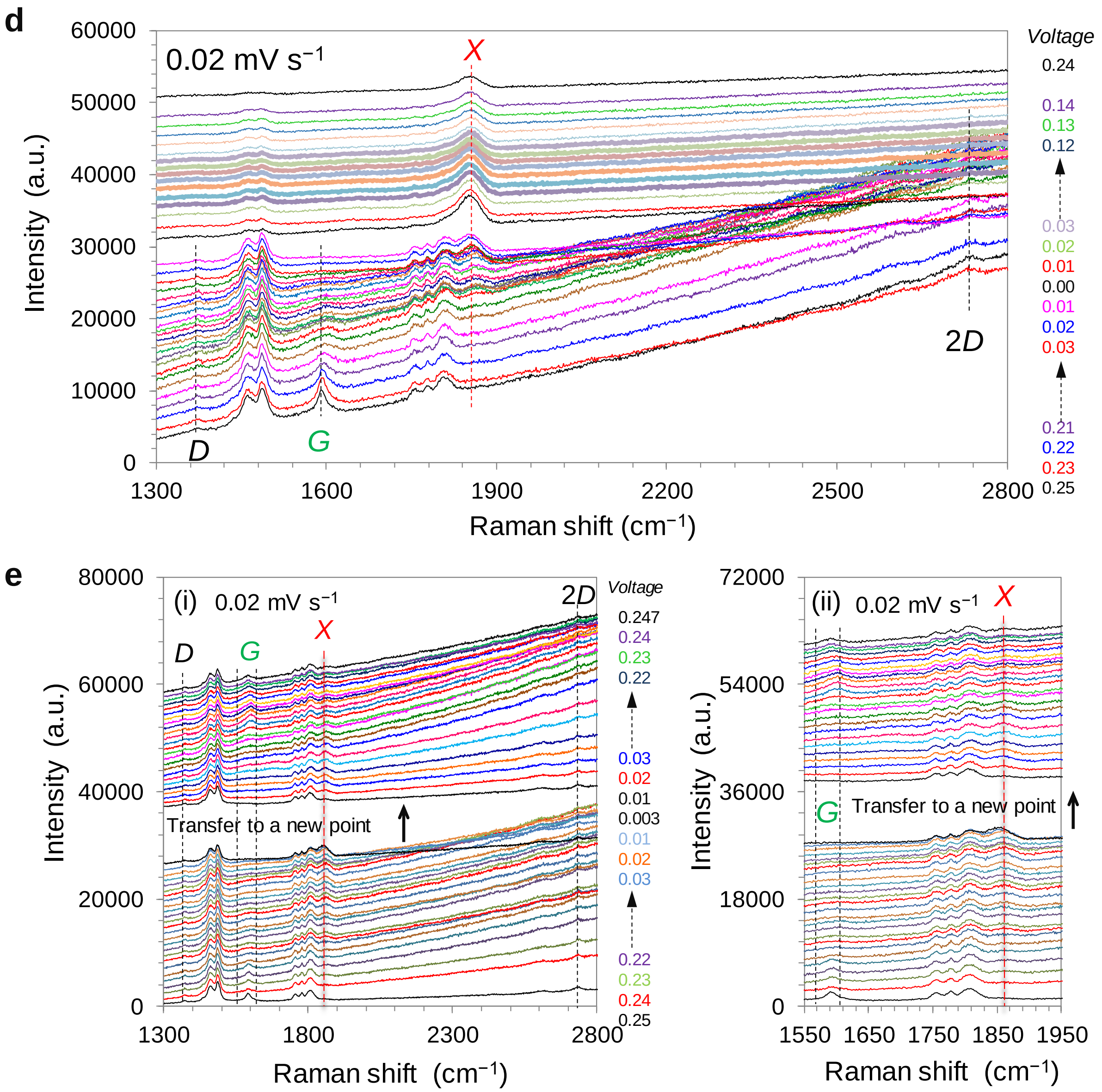

Supplementary Fig. 12 | *In-situ* Raman spectra for the graphene foam. d,**e**, *In-situ* Raman spectra with the CV rate at 0.02 mV s^{-1} (on behalf of a much longer laser-irradiation time than at 0.1 mV s^{-1}), which were captured at a fixed location (**d**) and then from two locations (**e**) in case of the irreversible irradiation damage suggested by (**d**). Each spectrum was recorded at an interval of 0.01 V. The *G* peak tended to vanish after 0.09 V during the Li intercalation process, and to reappear after 0.10 V during the Li deintercalation process, in line with the observation at 0.10 mV s^{-1} (**c**), which should be associated with the stacking configuration transformation between A*R*B and A*R*A induced by Li intercalation at this voltage range (corresponding to the phase changes from P3 to P2 and further PI). Both the failure illustrated in (**d**) and the rapid enlargement of the *X* band at a new probe position (see the spectra from 0.01 to 0.03 V in (**e**)) can further suggest the source of this special band associated with both the laser irradiation and Li concentration. Refer to the detailed discussion in Supplementary Note 1.

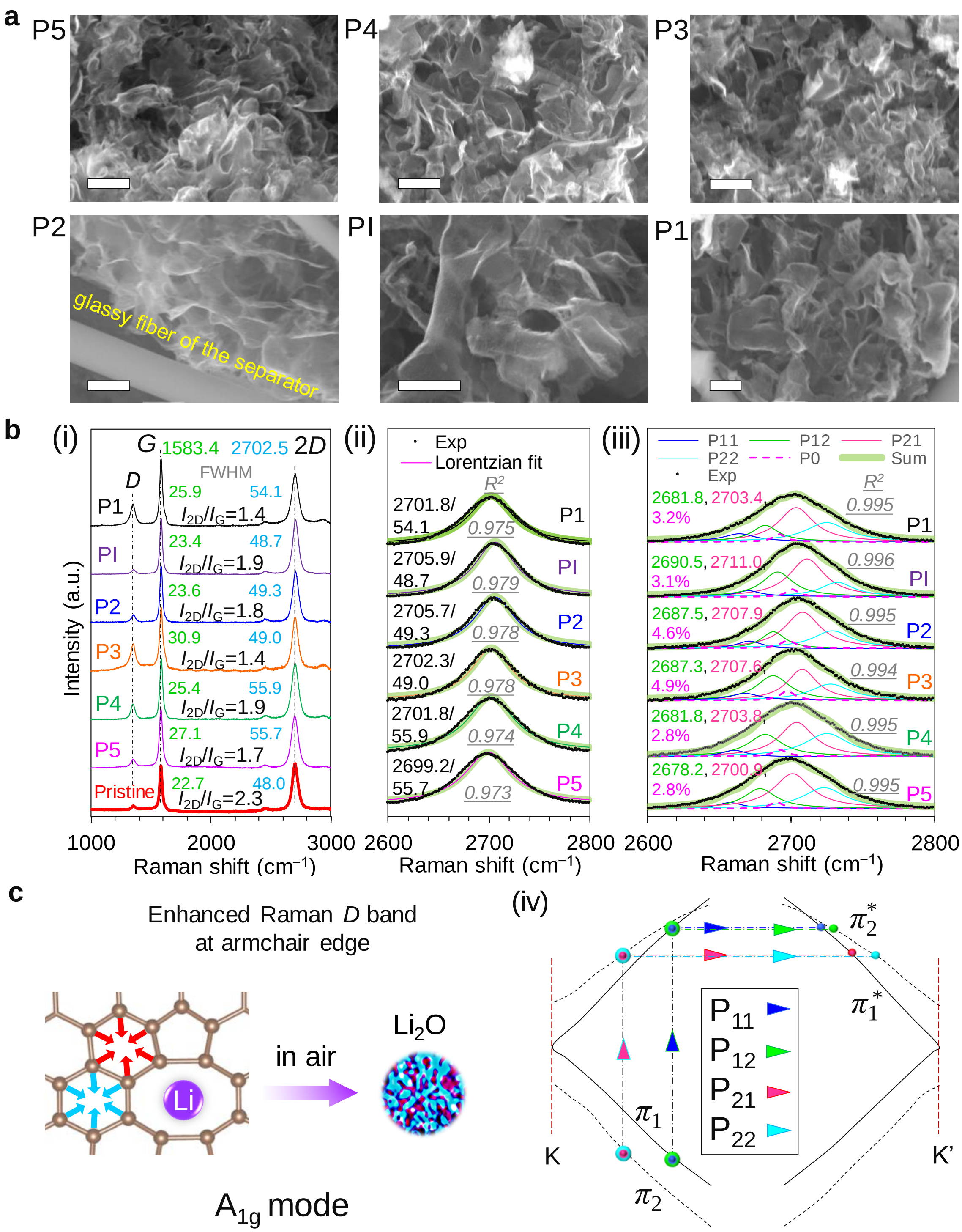

Supplementary Fig. 13 | Identification of the morphology and Raman features of the used bilayer-graphene foam electrodes. a, SEM images (scale bar, 1 μm) of the *ex-situ* Li-bilayer graphene samples at the P5, P4, P3, P2, PI, and P1 phases, respectively. The characterizations were carried out after the XPS measurement (Fig. 4 and Supplementary Fig. 21), when these lithiated samples had been oxidized due to being exposed in air for a long time. All the samples can maintain

the 3D porous morphology. **b**, Raman spectra of these samples (i) together with the comparative Lorentzian fitting analyses of their Raman 2*D* bands (ii−iv). (ii) Single peak-fitting results (with R^2 at 0.97−0.98), which simultaneously show the peak position and FWHM information (summarized in (i)). (iii) Combined-fitting results ($R^2 > 0.994$) by assuming that all the oxidized P3, P2, PI and P1 samples possessed the A-B stacking configuration (refer to the explanation below) and by using the same processing method in Fig. 1e(ii) (i.e., keeping the peak distance of P_{12} and P_{21} at 20−22 cm^{-1})[28–30], which display the contributions of monolayer or misoriented graphene regions (3−5% according to the integral area ratio of each pink fitting curve). (iv) Four scattering processes of the Raman 2*D* band of Bernal bilayer graphene corresponding to the P_{11}, P_{12}, P_{21} and P_{22} peaks in (iii)[29]. The assumption at (iii) about the stacking configuration of the oxidized P3, P2, PI and P1 was based on the following facts, such as their similar FWHM and I_{2D}/I_G values to those of the pristine Bernal bilayer graphene and the P5 and P4 oxidation products shown in (i), the significantly different intensity changes of the *in-situ* Raman *G* bands (Supplementary Fig. 12c−e), and the comparative XRD results for the fresh and oxidized lithiated samples (Supplementary Fig. 16). Accordingly, although the macroscopic morphology (like porous structure) of the oxidized P3, P2, PI, and P1 samples (with relatively higher Li distribution densities) maintained as well as those of P5 and P4 (**a**), the oxidation and aggregation of Li atoms during the air exposure possibly caused all the oxidation products to possess the microscopic A*R*'B stacking configurations (*R*': the generated Li_2O_x layer). Thus, there should be two reasons which can account for the differences between the *ex-situ*[31] and *in-situ*[2] Raman spectra focusing on the intensity and shift of *G* band, including here the extrinsic configuration change caused by Li oxidation and the inherent Li-doping effect[2,32,33] discussed at Supplementary Fig. 12. **c**, Schematic of the influence of Li atoms at the defective sites (e.g., DVs) on the Raman *D* band intensity enhancement[34]. The Raman *D* band is known to be generally induced by the A_{1g} zone-boundary mode at the graphene edge[34], including the equivalent defective sites of high order illustrated in (**c**). Therefore, during the *in-situ* experiment (Supplementary Fig. 12), because Li^+ ions just diffuse through these sites and Li atoms simply adsorb there, the quality of graphene sheet won't be disrupted to exhibit enhanced *D* band. However, once the lithiated graphene samples were exposed in air (the cases here), Li atoms would be oxidized into larger Li_2O_x species at the defective sites to greatly aggravate these defects, which seemed to be particularly obvious at high Li-density but structurally unstable phases[31] like P3 and P1 (refer to the HR-TEM and XPS results in Supplementary Figs 14 and 21b, respectively).

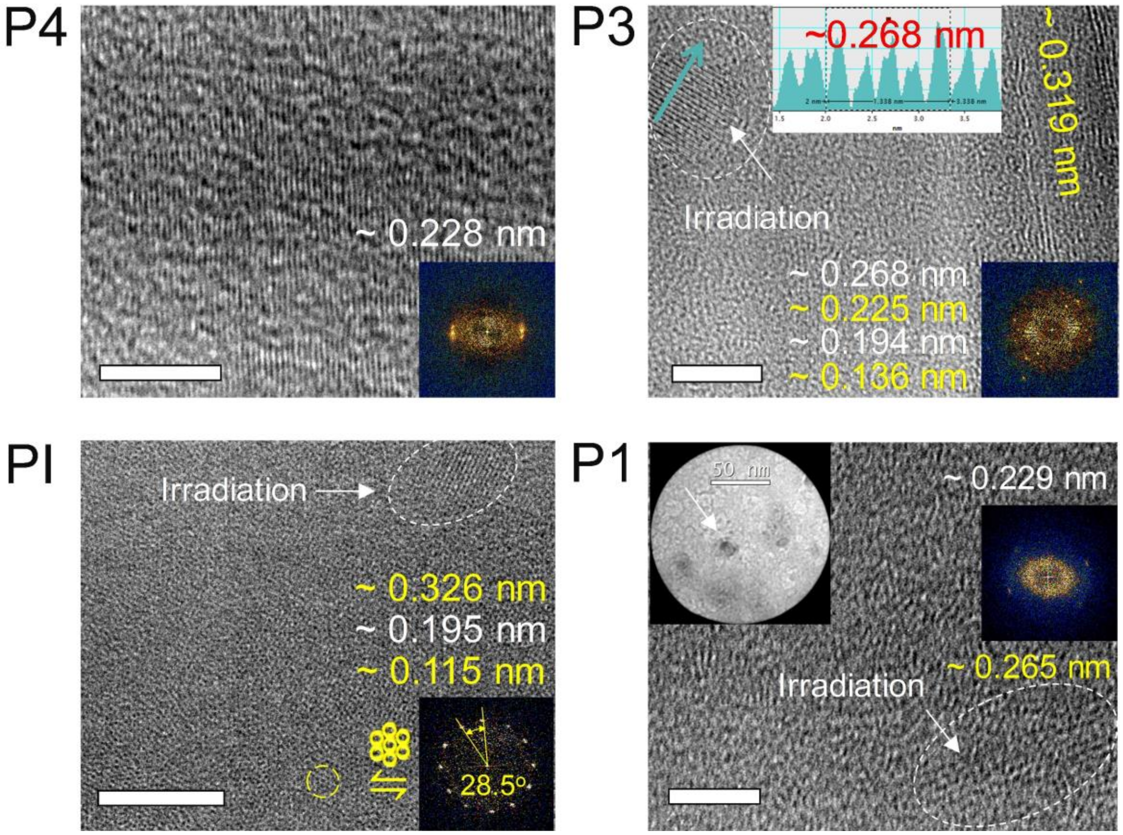

Supplementary Fig. 14 | Typical HR-TEM images of the well-preserved P4, P3, PI, and P1 samples. Scale bar, 5 nm. The lattice spacings were all estimated by using the inset Fast Fourier Transform (FFT) patterns, and the white-color values refer to the dominant lattice sizes. The P3 and P1 samples displayed relatively blurry fringes possibly due to the unstable stacking configurations of the selected local areas under irradiation. The PI image displayed a FFT pattern composted by two sets of hexagonal patterns[13] with a rotational angle of ca. 28.5° (characteristic of quasicrystalline bilayer graphene[35,36]), which was quite similar to its SAED pattern collected at the same but much enlarged region (Supplementary Fig. 17c(iv,v)). The spacing at ca. 0.32 nm may come from the DV sites. The spacings of 0.27 and 0.23 nm are possibly from the inflated honeycomb lattice constants, when Li atoms driven by electron beam would enter into the hexagonal centroids (refer to the configuration model in Fig. 3b(iv)). Besides, the (002) spacing of LiF (possibly present in the SEI film, $LiPF_6 \rightarrow LiF + PF_5$) is ca. 0.190 nm[37], which may disturb the localized HR-TEM imaging but it possesses no sixfold-symmetry electron diffraction pattern. In brief, quite different from the pristine bilayer graphene (Supplementary Fig. 3), these lithiated graphene phases displayed discriminable lattices in their HR-TEM images. Besides, we noticed that prolonging irradiation time would enable the lattice fringes to become much clearer (refer to the circle-marked local regions in P3, PI, and P1), suggesting the considerable influence of electron beam (refer to the SAED results in Fig. 3a–c and Supplementary Fig. 17 as well as the above laser-irradiation damage in Supplementary Fig. 12d). The commensurate rotation of the two graphene layers for PI should also be associated with the irradiation effect (refer to Supplementary Fig. 18b).

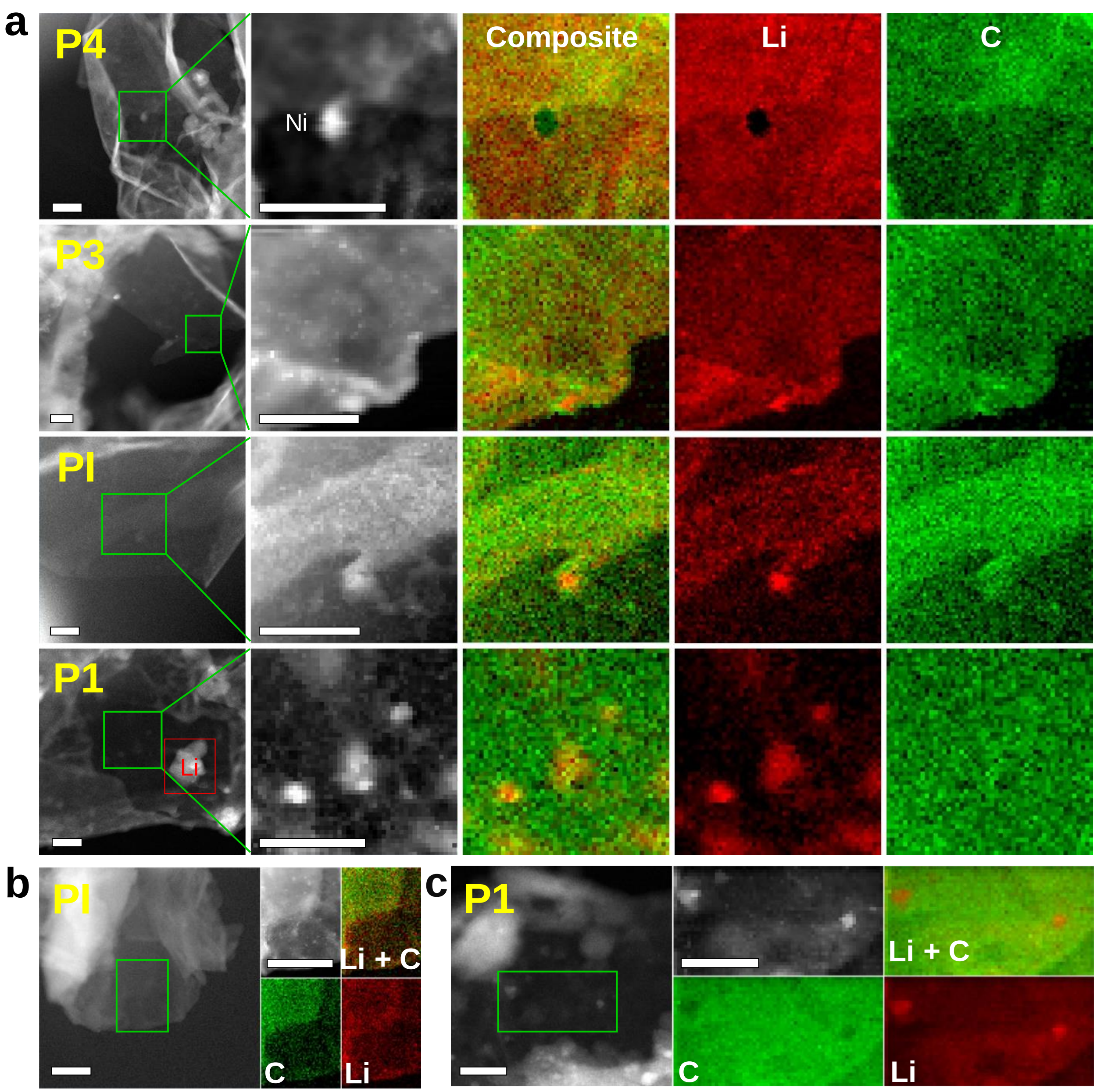

Supplementary Fig. 15 | EELS analysis. a, Comparative EELS mappings for the well-preserved P4, P3, PI, and P1 samples. **b**,**c**, EELS mappings captured from another regions for PI and P1. All the scale bars refer to 100 nm. No Li dendrites generated in all the cases, except that some big Li particles or clusters appeared on the basal plane of the sole P1 sample (**a**, **c**). These results suggest the defective or edge regions (including the junction of fewlayer and bilayer areas shown by PI in (**a**)) of graphene sheets to induce the Li enrichment (or called Li-plating behavior[38]) and thus the Li-storage position (i.e., graphene interlayer). The contrast evolution displayed by the composite images (**a**) may be associated with the staged SEI on the surface (refer to the discussions about the *in-situ* Raman spectra and O 1s XPS results in Supplementary Figs 12 and 21, respectively).

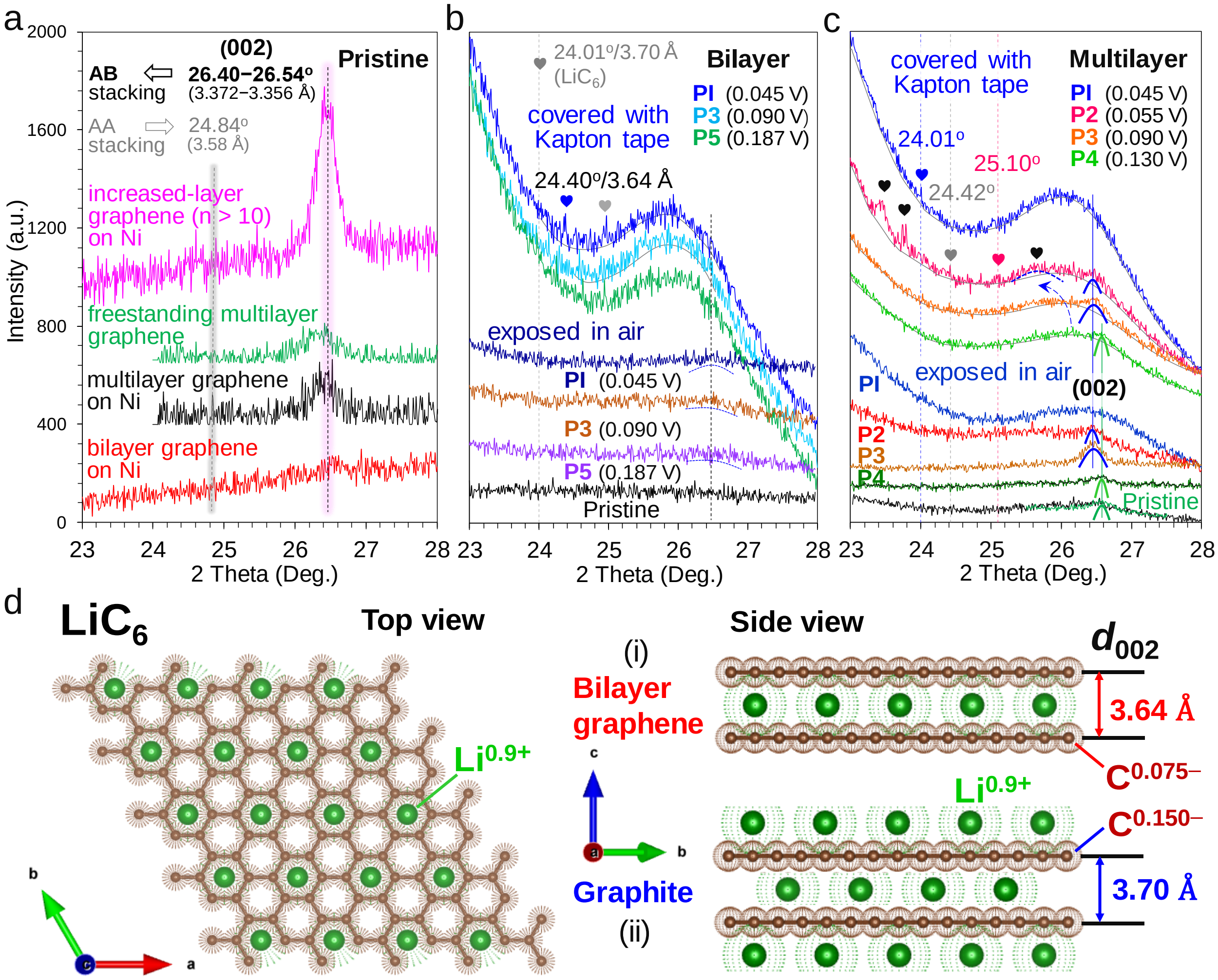

Supplementary Fig. 16 | XRD analysis to identify the interlayer spacing information. a, XRD patterns of pristine graphenic carbon foams with increased graphene layers (until the average *n* value > 10). All the pristine graphene foam samples should possess the predominant AB stacking configuration in view their larger 2θ angles (with the typical interlayer spacings d_{002} at 3.36 ± 0.01 Å) than that for AA stacking (24.8°, d_{002} = 3.58 Å)[39], in line with the Raman results and discussion (Fig. 1e). The slight shift of the XRD (002) peak for the bilayer and multilayer samples (also considering the reported varied d_{002} values for different graphites, 3.34−3.37 Å) may result from the altered fractions of their coexisting misorientation configurations. The (002) peak of the bilayer sample (as well as its lithiated products (**b**)) is rather weak due to the sole crystal face for diffraction (**d**(i)). **b**, XRD patterns of the bilayer graphene-based samples before and after being oxidized/exposed in air. Kapton tape displays no sharp XRD diffraction peak. In view of the patterns of the three fresh bilayer products, a new peak seemed to emerge at 24.4° at the PI phase (with A*R*A configuration in theory). Although its corresponding d_{002} value (3.64 Å, if correct) was smaller than that for LiC_6 of graphite electrode (~3.70 Å, 24.0°), it is understandable by considering the different average valences of C atoms in C_6LiC_6 (bilayer graphene) and LiC_6

(graphite) and thus the varied repulsive forces between the adjacent graphene sheets (refer to (**d**) and the smaller d_{002} for AA stacking graphite). After exposure to air, however, each oxidation product seemed to display a sole diffraction peak at 26.5° (no LiC_x peaks), as further confirmed by the stronger-signal spectra of the oxidized multilayer samples (**c**). This specific phenomenon should indicate that, all the oxidation products possessed the A*R*'B structure (*R*': Li_2O_x layer) regardless of the intrinsic stacking configurations of their fresh counterparts, possibly due to the random oxidation and thus aggregation of Li atoms at the interlayer on this occasion, in favour of the Lorentzian fitting analyses of the *ex-situ* Raman spectra (Supplementary Fig. 13b). **c**, XRD patterns of the multilayer graphene-based samples before and after being oxidized/exposed in air. The XRD pattern of the pristine sample was double checked, from which its (002) diffraction peak was determined to be centred at 26.59° (d_{002} = 3.35 Å). In view of the typical patterns of the overall four fresh lithiated samples, as the Li concentration increased, this original (002) peak was slightly blueshifted in the foregoing phases until 26.42° at P2 (d_{002} = 3.37 Å) and seemed to vanish at the final A*R*A-stacking PI, exhibiting a suspected new peak at 24.01° (d_{002} = 3.70 Å, quite close to that for LiC_6). The pattern of the material for the transitional P2 phase (refer to the highly overlapping CI and C2 CV peaks in Fig. 2b and Supplementary Fig. 8c and to the abnormal Li 1s and C 1s XPS spectra of P2 in Fig. 4a,c) was quite similar to that of the unstable LiC_{12} stoichiometry of graphite (identified to be a mixture of LiC_6 and "liquid-like" LiC_{12-18})[22], which displayed multiple diffraction peaks such as at 23.5° (3.78 Å), 23.8° (3.74 Å), 24.4° (3.64 Å), 25.1° (3.54 Å), and 25.7° (3.46 Å). In line with the P2 pattern, there was also a broad diffraction peak centered at ca. 25.7° (3.46 Å) for P3 in addition to the retentive one at 26.4° (refer to the stronger and broader (002) peak of its oxidation product). In view of the definite LiC_x composition at P3 for either graphenes or graphite (different from that at P2), we consider its real product to possibly form two stacking configurations, including the initial A*R*B mode and another relatively balanced/stable SP mode (namely a theoretically intermediate state between A-B and A-A, refer to Fig. 4e,f and Supplementary Figs 6c,d and17b(xi)), consistent well with its Li 1s XPS spectrum containing two Li 1s species (Fig. 4c). Thus, P3 can be regarded as another transition phase due its relatively large Li density (refer to its multiply peculiar results summarized at Supplementary Fig. 19); however, due to its much lower Li density, it is more stable than P2 (possibly with a theoretically quasi-SP structure by following P3, and considering the ideal SP structure of either $C_{14}LiC_{14}$ for P3 (Scheme 1b) or C_6LiC_6 for P1 (Fig. 4e,f)). The single weak peak of 26.6° for P4 indicates that this low-Li density phase should retain the A-B structure of the pristine sample, and so should the P5 phase. Even so, the intercalated Li atoms seems to be able to be located at two sites at P4 (namely Site 1' and Site 2' shown in Fig. 4e,f) in its ideally stacking configuration (Scheme 1b and Supplementary Fig. 9b), still agreeing with its C1s, Li 1s, and O 1s XPS results and further explaining the difference between its XPS spectra and those of P5 (Fig. 4a−d and Supplementary Fig. 21b−d). **d**, Schematic of the Z-axis structures of the PI phases (with LiC_6 stoichiometry in plane) for (i) bilayer graphene and (ii) graphite/multilayer graphene, assuming that the PI pattern in (**b**) is accurate and the valences of the embedded Li atoms are constant at +0.9 (or with a quite slight change)[3,40,41].

a

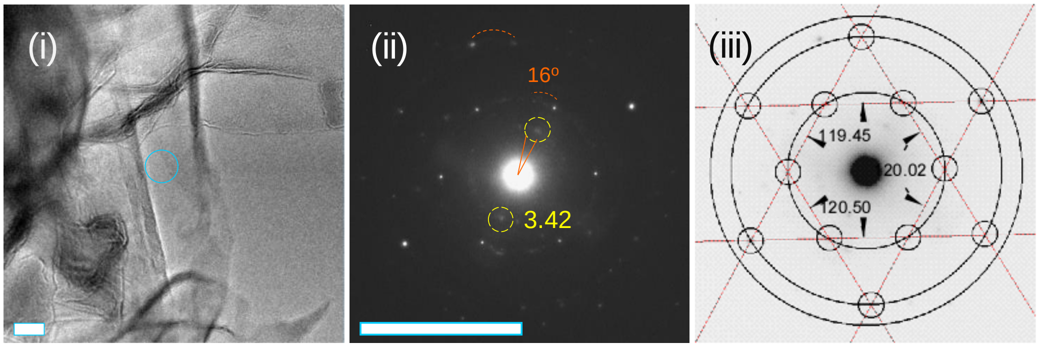

Supplementary Fig. 17a | Normal-incidence SAED pattern from a flat region for the bilayered P4 sample. The scale bars refer to 50 nm and 1 Å^{-1} in (**i**) and (**ii**), respectively. The angles close to 120°, together with the high coincidence of the circles and regular hexagon connecting the target {0110} and {1210} diffraction spots (**iii**), can help confirm that, the as-obtained pattern was captured from a flat enough region (**i**). Thus, it is feasible to use the data for the further analysis, as shown in Fig. 3a(i) for the brightness ratio of the {0110} and {1210} peaks (ca. 0.53) and in Fig. 3c for their corresponding in-plane lattice spacings (ca. 2.12 and 1.23 Å). Similar processing method (geometrical analysis) was applied to identify the SAED patterns of the other samples. As a result, the interplanar distance was estimated to be about 3.42 Å from the weak diffraction ring in this pattern, a little larger than the theoretical d_{002} value at this low-Li density phase (ca. 3.35−3.37 Å at the A*R*B configuration by the XRD results in Supplementary Fig. 16). In consideration of here the locally misoriented stacking configuration caused by the beam irradiation (as suggested by the rotation angle marked in this pattern, refer to Fig. 3a,b and Supplementary Figs 18 and 19) and the typical interplanar spacings for graphite (varied between 3.35 Å at AB mode and 3.58 Å at AA mode)[39], the d_{002} value for the A*R*B-stacking P4 (with low Li concentration) is supposed to be smaller than the observed 3.42 Å in (**ii**) and be more close to 3.35 Å.

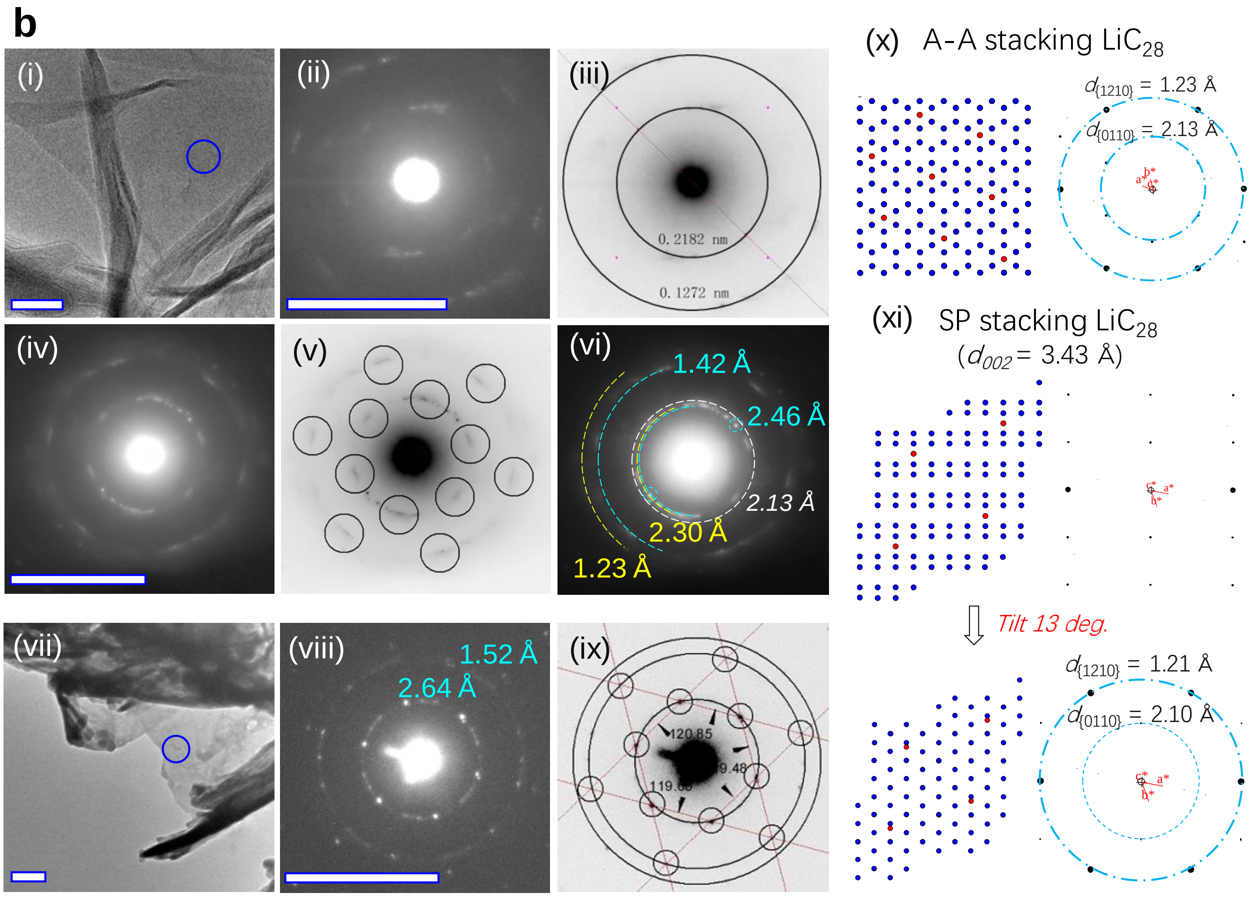

Supplementary Fig. 17b | Normal-incidence SAED patterns of the bilayered P3 sample and simulated in-plane spacings for the given LiC_{28} structure models with varied stacking orders. The scale bars refer to 50 nm in (**i**), 200 nm in (**vii**), and 1 $Å^{-1}$ in (**ii**, **iv**, **viii**), respectively. The patterns in (**ii**; i.e., Fig. 3a(ii)) and (**iv**) were captured rapidly and successively from the same region marked in (**i**), and the pattern in (**viii**) was obtained at another position (**vii**) after long-time irradiation. The initially sixfold symmetry for the {0110} and {1210} peaks became rather ambiguous at this phase, as further confirmed by using one multilayered sample in Supplementary Fig. 17e (refer to the literature statement about this phase of graphite, namely no in-plane ordering[19,24,42,43]). The ring-like diffraction patterns (**ii**, **iv/vi**, **viii**) should be composed by several hexagonal diffraction patterns with arbitrary rotation angles (refer to (**viii**)), reflecting various incommensurate/misoriented stacking configurations. In the initial pattern (**ii**, **iii**), the two in-plane spacings were measured to ca. 2.18 and 1.27 Å (ca. 3% larger than the pristine values). However, there appeared four spacings in the later pattern with more identifiable diffraction spots (**iv**−**vi**). Thereinto, the {0110} spacing increased to 2.298 Å (ca. 7.9% larger) from the normal 2.13 Å (denoted by the white ring in (**vi**)), in line with the above HR-TEM observation (Supplementary Fig. 14) and the theoretical expansion degree for the honeycomb lattices of the Li-inlaid LiC_{28} (Supplementary Fig. 19b), while the {1210} spacing (ca. 1.232 Å) still seemed to approach to that of pristine graphene. Another two spacings in (**vi**) were ca. 2.463 and 1.422 Å, rather close to those (2.464 Å and1.426 Å) of the lattice constant and C-C bond length for pristine graphene (refer to

Fig. 3a(iv))[38,40]. In the third pattern (**vii**−**ix**), the lattices spacings were measured to be 2.641 and 1.518 Å from the highly distinguishable diffraction spots with sixfold symmetry (refer to Fig. 3b(iv)). Quite similar to the above expansion degree of the {0110} planes, these two values were 6−7% larger than the initially lattice constants assigned to the honeycomb units of graphene. The simulation results in (**x**, **xi**) can help account for the above experimental results under different-degree irradiation (similarly in the Supplementary Figs 17c(x), 18, and 19). Moreover, it can be seen that the simple changes in the interplanar spacing (e.g., with d_{002} at 3.33, 3.43, and 4.00 Å), stacking configuration[11,12], and Li density would not influence the in-plane lattice spacings. All the above phenomena, including the initial ambiguous patterns (**ii**), the appearance of two sets of diffraction spacings after short-time irradiation (**vi**), the multiple sets of diffraction patterns after adequate irradiation (**viii**), and the ever-increasing lattice spacings, were considered to be induced by the beam irradiation (refer to the schematic explanation in Supplementary Figs 18 and 19). In short, the high-energy electrons should easily drive the interbedded Li atoms (considering their "real" valence states in the graphene interlayer, e.g., +0.9|e| for Li in the C_6-Li constitutional unit due to the charge transfer between it and its surrounding C atoms)[3,40,41] to enter into the hexagonal centroids of one graphene layer (denoted as Li-inlaid graphene layer below), leaving the other layer to become a "pure"/Li-free graphene sheet. The random movements of electrons and Li atoms (refer to Supplementary Fig. 19a) would further exacerbate such misorientation in the stacking order (incommensurate structure) between the lattice-expanded Li-inlaid sheet and the pure graphene one (refer to Supplementary Fig. 18).

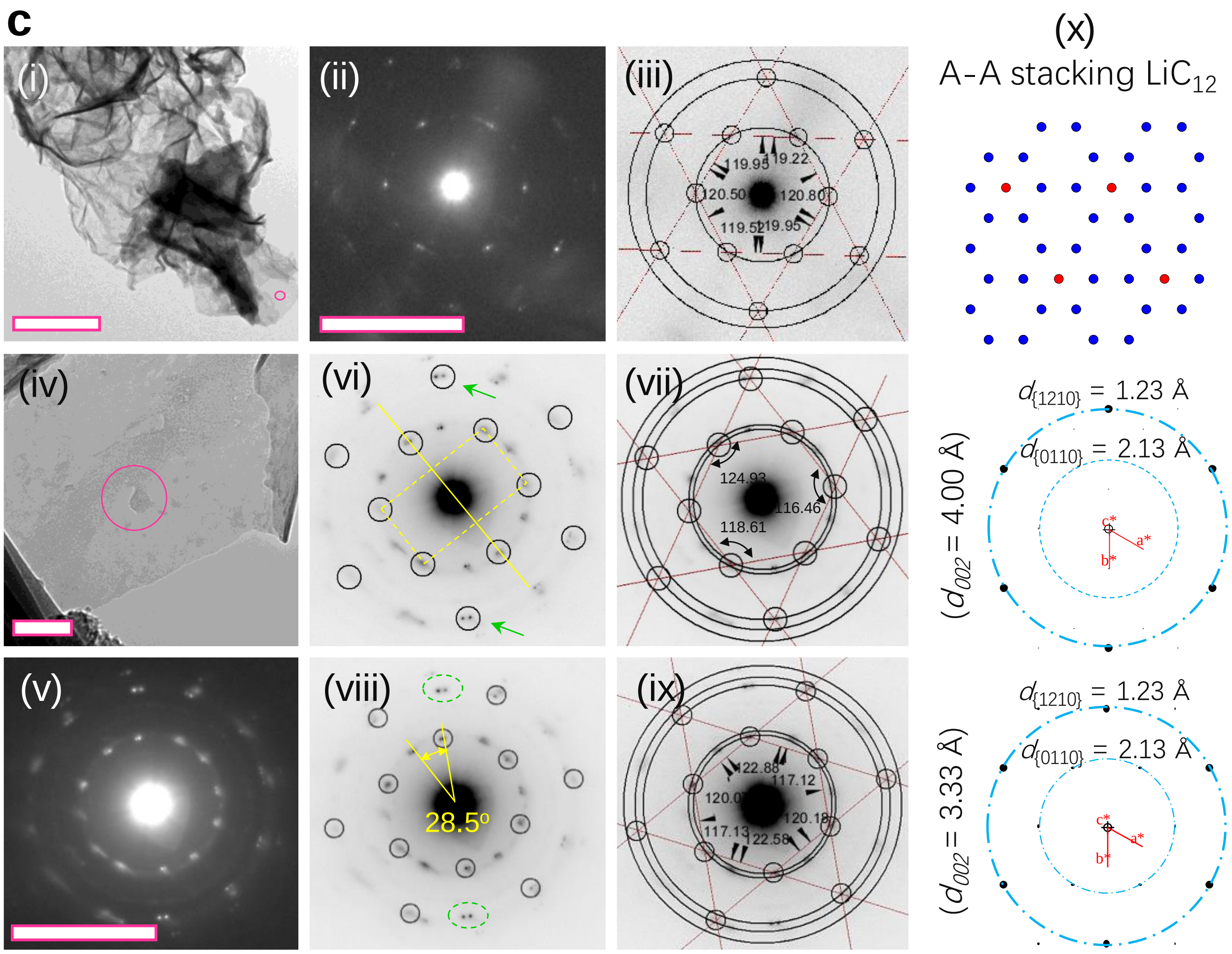

Supplementary Fig. 17c | Normal-incidence SAED patterns of the bilayered PI sample and the simulated in-plane spacings for the A*R*A-LiC_{12} models with varied interplanar spacings. The scale bars refer to 500 nm in (**i**), 100 nm in (**iv**), and 1 Å^{-1} in (**ii**, **v**), respectively. The pattern in (**ii**, namely Fig. 3a(iii)) was captured rapidly, when the in (**v**) one was obtained by prolonging irradiation time. The EELS results in Supplementary Fig. 15a indicated the coexistence of fewlayer graphene at the sampled area in (**iv**). From the orthohexagonal pattern in (**i**–**iii**), the lattice spacings of the {0110} and {1210} peaks were estimated at 2.11 and 1.22 Å (marked by the violet circles in Fig. 3c), respectively. The mixed pattern in (**iv**, **v**), displaying two sets of diffraction spots (with a rotation angle of 28.5^{o} (**viii**), refer to Supplementary Fig. 14 and the similar observation at the P1 phase in Supplementary Fig. 17d(vii)), possibly suggested the misorientation of the adjacent graphene layers at this sampled flat area[13]. The paired/parallel diffraction spots (marked by green in (**vi**, **viii**)) indicated that this Li-graphene region was also perhaps a little curved[12] (refer to the inner angles in (**vii**, **ix**) and Region 1 in Supplementary Fig. 3e). The two distorted sets suggested the in-plane lattice spacings to be ca. 2.19/1.27 Å (ca. 3% larger) and 2.06/1.19 Å (ca. 3% smaller), corresponding to the black and pink symbols in Fig. 3c, respectively. All the phenomena in (**iv**–**ix**) can be well explained by the irradiation effect discussed in detail in Supplementary Fig. 18.

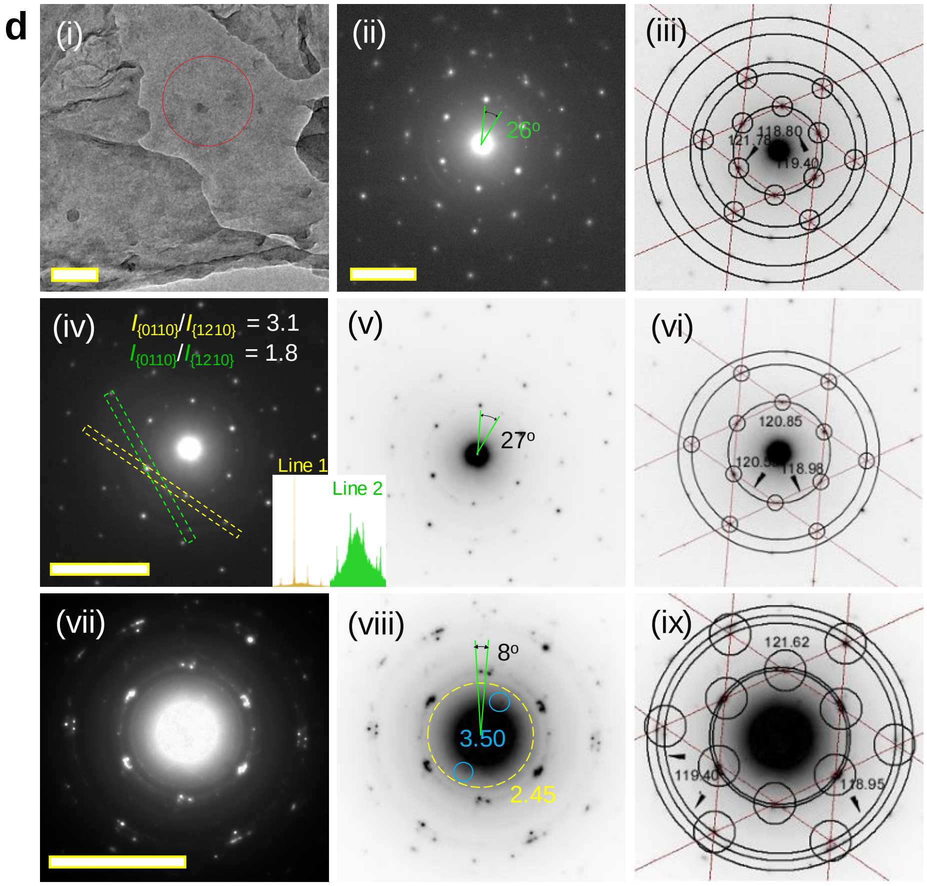

Supplementary Fig. 17d | Normal-incidence SAED patterns of the bilayered P1 sample after some-time irradiation. The scale bars refer to 100 nm in (**i**) and 1 Å^{-1} in (**ii**, **iv**, **vii**), respectively. These patterns, including Fig. 3b(iii), were captured successively from the large flat region marked in (**i**, refer to the HR-TEM result in Supplementary Fig. 14). The $I_{\{0110\}}/I_{\{1210\}}$ ratios for the bright/dark paired patterns (marked by yellow/green in (**iv**)) were measured to be 5.0/1.9 in (**ii**), 3.1/1.8 in (**iv**), and 2.1/1.8 in Fig. 3b(iii), respectively. The in-plane lattice spacings of the {0110}/{1210} peaks (summarized in Fig. 3c) were about 2.18/1.25 Å (ca. 2% larger) in (**ii, iii**), 2.09/1.20 Å (ca. 2% smaller) in (**iv**−**vi**), and 2.18/1.25 Å (ca. 2% larger) mixed with 2.07/1.18 Å (ca. 2−4% smaller) in (**vii**−**ix**), respectively. A spacing (2.45 Å) close to the lattice constant of graphene (2.46 Å) was also observed in (**viii**), similar to that at the P3 phase (Supplementary Fig. 17b(vi)). The d_{002} interplanar spacing observed from (**viii**) was 3.50 Å, much smaller than the value indicated by the nondestructive XRD technique (3.64 Å if not reaching 3.70 Å of LiC_6; Supplementary Fig. 16b−d). The reason for this result should lie in the beam irradiation or rather the incommensurate structure it caused (refer to the discussion at Supplementary Fig. 17a), as reflected by both the above different in-plane lattice spacings of the pure and Li-inlaid graphene layers and their stacking order deviating from the original A*R*A mode (ca. 8° in the rotation angle here; see the extrinsic structure model in Supplementary Fig. 18b). Such consistently physical phenomena induced by the electron beam irradiation further verify the C*R*C stacking configuration of the original Li-bilayer graphene phases.

e

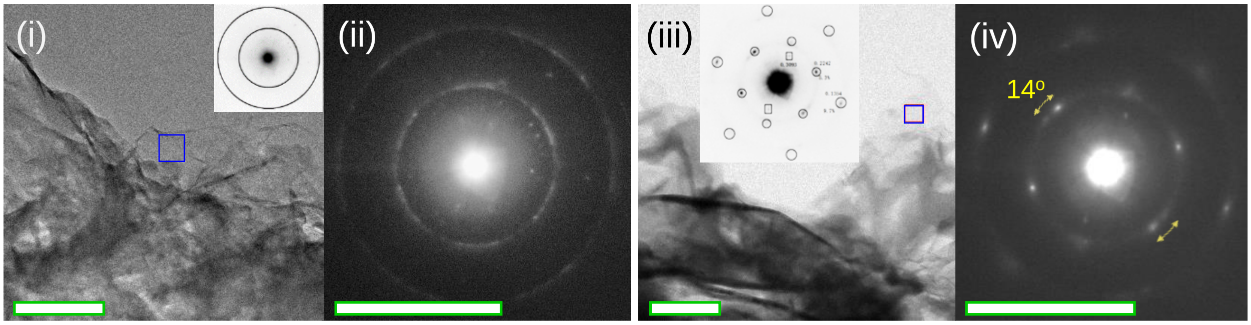

Supplementary Fig. 17e | Normal-incidence SAED patterns of the multilayered P3 sample. The scale bars refer to 200 nm in (**i**, **iii**) and 1 Å^{-1} in (**i**, **iv**), respectively. The long-time electron beam irradiation caused the ring-like diffraction pattern (**i**, **ii**) to become a distinguishable orthohexagonal pattern (**iii**, **iv**), in line with the bilayered phenomena (Supplementary Fig. 17b), indicating the relative rotating between graphene layers driven by the chaotic movements of the intercalated Li atoms (Supplementary Fig. 19a). The in-plane spacings for the {0110} and {1210} peaks also became lager in this sample, e.g., 3−6% for the enhanced {0110} peaks (with d_{0110} at ca. 2.19 and 2.24 Å in (**ii**) and (**iv**), respectively). In addition, the observed area was noticed to roll up at the end (refer to the phenomenon in Supplementary Fig. 17c(vi,viii)), indicating varied strains between graphene layers with Li embedding or not (Supplementary Fig. 18b).

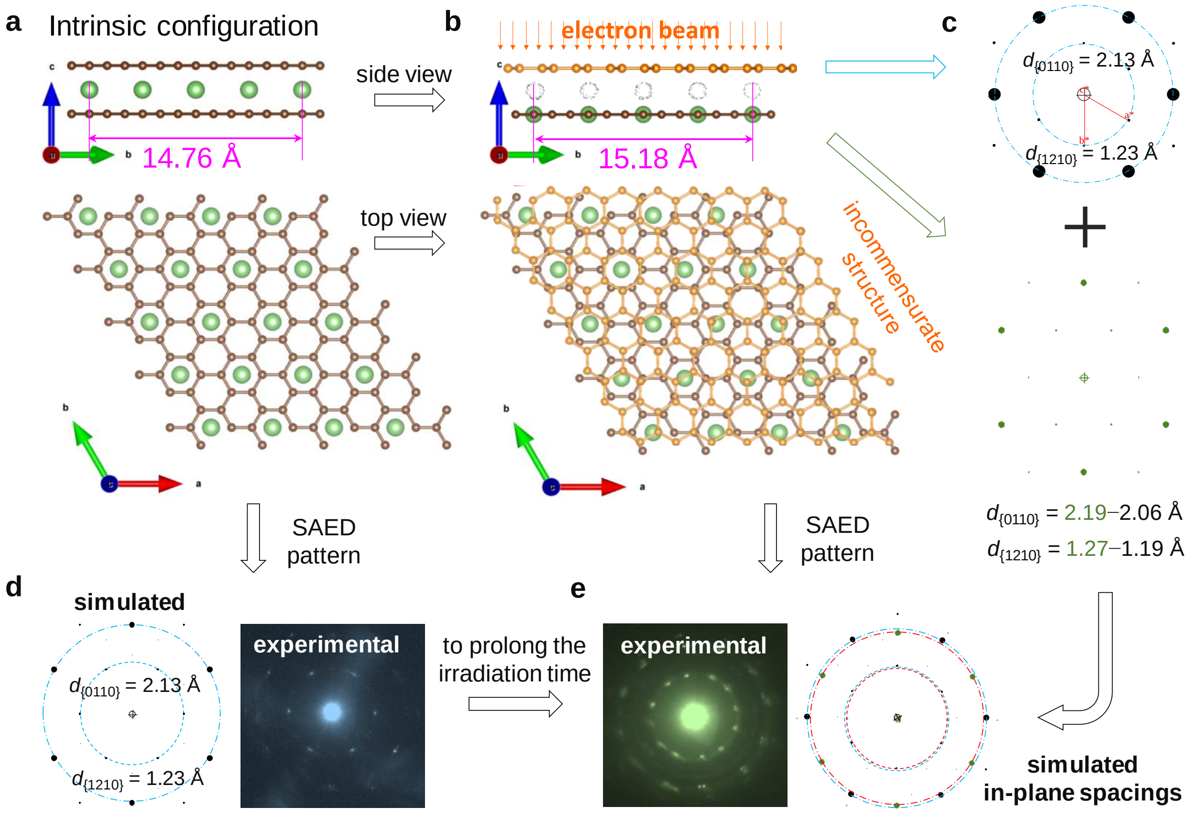

Supplementary Fig. 18 | Interpretation of the relationship between the beam irradiation and the SAED patterns at PI. a,**b**, Schematic illustration of the structural remodeling of the Li-saturated bilayer graphene (namely C_6LiC_6) under the electron beam irradiation. **c**–**e**, Theoretical and experimental SAED patterns of C_6LiC_6 before and after the configuration transformation. The DFT-based structural relaxation shows that, as Li atoms enter into the hexagonal centroids of graphene sheet, the lattice constant would expand by 6.5% for stoichiometric LiC_{14} (Supplementary Fig. 19b) and by 3% for stoichiometric LiC_6 (refer to the bottom layer in (**b**) and its simulated diffraction pattern in (**c**)), in good agreement with the experimental observations (Supplementary Fig. 17c,d). Although such a process is impossible in practical LIBs due to the high formation energy (~4.16 eV for Li atom in LiC_{28} by our DFT calculation)[3], it should be feasible under the TEM condition based on the above systematic study. The high-energy electron beam can easily drive the interlayered Li atoms to enter into honeycomb lattices of the bottom sheet of bilayer graphene (**b**, side view). As a result, besides of the possible commensurate rotation (with an arbitrary/indeterminate angle)[13], the lattice expansion in the Li-inlaid graphene sheet would lead it to possess obviously incommensurate structure to that of the upper Li-free graphene sheet (**b**), yielding the mixed SAED patterns (**c**, **e**; refer to the direct phenomena in Supplementary Figs 14 and 17) consisting of two main hexagonal patterns with more obvious monolayer feature (refer to the larger $I_{\{0110\}}/I_{\{1210\}}$ ratios shown in Fig. 3b and Supplementary Fig. 17d). Meanwhile, the interplanar spacing would possibly decrease along with the structural remodeling under electron beam. Consequently, to avoid/alleviate the irradiation interference, it is a must to capture the SAED pattern as quickly as possible (Fig. 3a).

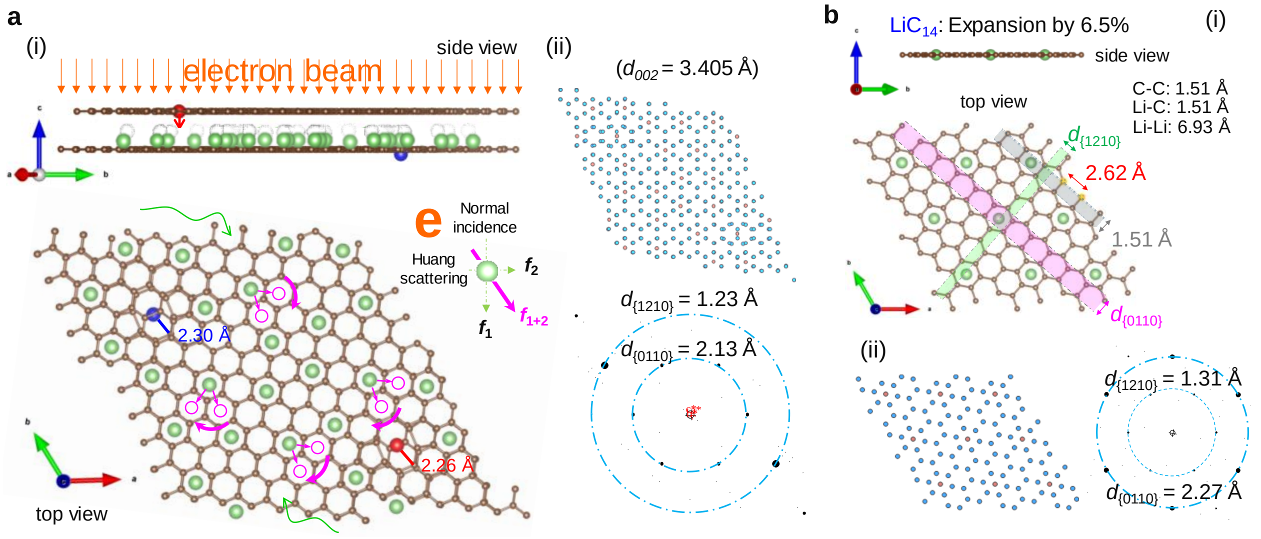

Supplementary Fig. 19 | Interpretation of the influence of the electron beam irradiation on the specialized SAED patterns at the P3 phase. a, Schematic of the influence of the vacancies (refer to the purple circles) on the electron beam-driven movements of Li atoms in the Li-unsaturated bilayer graphene. For clarity, A*R*A stacking configuration (**i**) is adopted as the initial state in the current model, whose simulant diffraction pattern (static) is shown in (**ii**). Besides, the defect area (represented as DV sites here) is ca. 3% and the C/Li atom ratio is 17.6. Because of the abound vacancies in the 2*D* space, the interlayered Li atoms can not only move freely in the Z-axis direction but also on the X-Y plane. The chaotic/unsynchronised motions of a sea of Li atoms driven by electrons with large momentum (**i**) will possibly lead regional graphene sheets (at the bottom layer relative to the incident direction of the electron beam) to rotate in an arbitrary angle, giving rise to various incommensurate stacking configurations in the selected area and thus the ring-shape SAED pattern like that for a polycrystalline material/region (refer to Region 2 in Supplementary Fig. 3e). This phenomenon can be identified in all the samples (Fig. 3a and Supplementary Fig. 17) especially the "liquid-like" P3 phase (Supplementary Fig. 17b,e). By the way, such a Li-density state, large enough but yet-to-be saturated in the graphene interlayer, may also account for the other peculiar characterization results at P3 (under different disturbances in the experimental processes), such as the incompletely reversible CV behavior (Supplementary Fig. 6c,d), the zigzag Nyquist plot (Supplementary Fig. 10b), the unordered lattice fringes (Supplementary Fig. 14), and so on. In addition, although the presence of DV sites (with the maximum Li-C distance at ca. 2.3 Å[3,40]) will facilitate this unsteadiness under electron beam irradiation, such local bonding would not contribute to Bragg peak in the diffraction pattern (**ii**) but only diffuse scattering (i.e., Huang scattering shown in (**i**)). **b**, Structure model for Li-inlaid monolayer graphene (LiC_{14} stoichiometry) with lattice expansion at 6.5% (**i**) and its corresponding diffraction pattern (**ii**), consistent well with the experimental result in Supplementary Fig. 17b(viii).

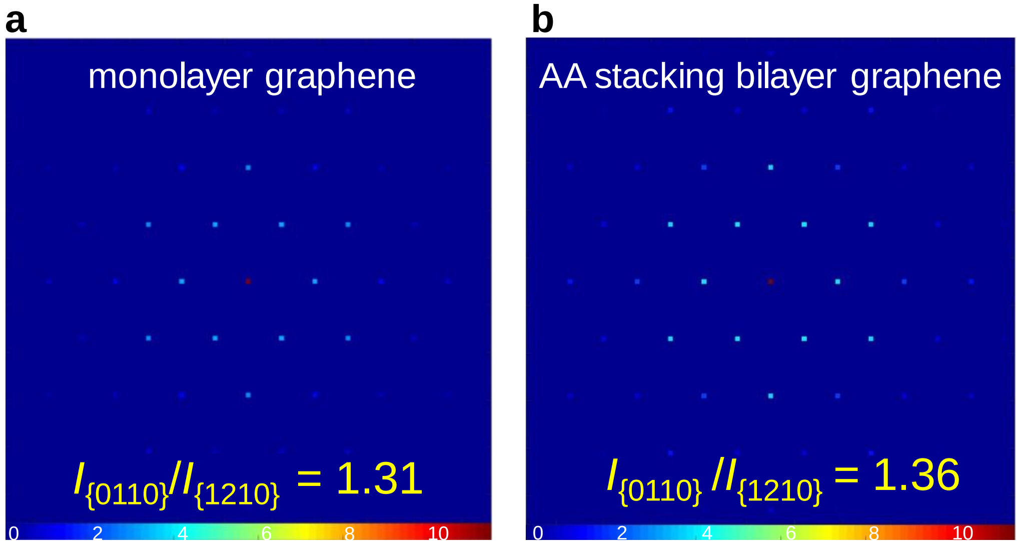

Supplementary Fig. 20 | Brightness simulation for the electron diffraction patterns of graphenic carbons. a, Monolayer graphene. **b**, AA stacking bilayer graphene. The results for either monolayer graphene (**a**) or AB stacking bilayer graphene (Fig. 3d) are quite close to the literature values (~1.4 and 0.4–0.5, respectively)[11,15], validating our SAED simulation approaches (refer to the detailed brightness values of the {0110} and {1210} diffraction spots in Supplementary Table 1).

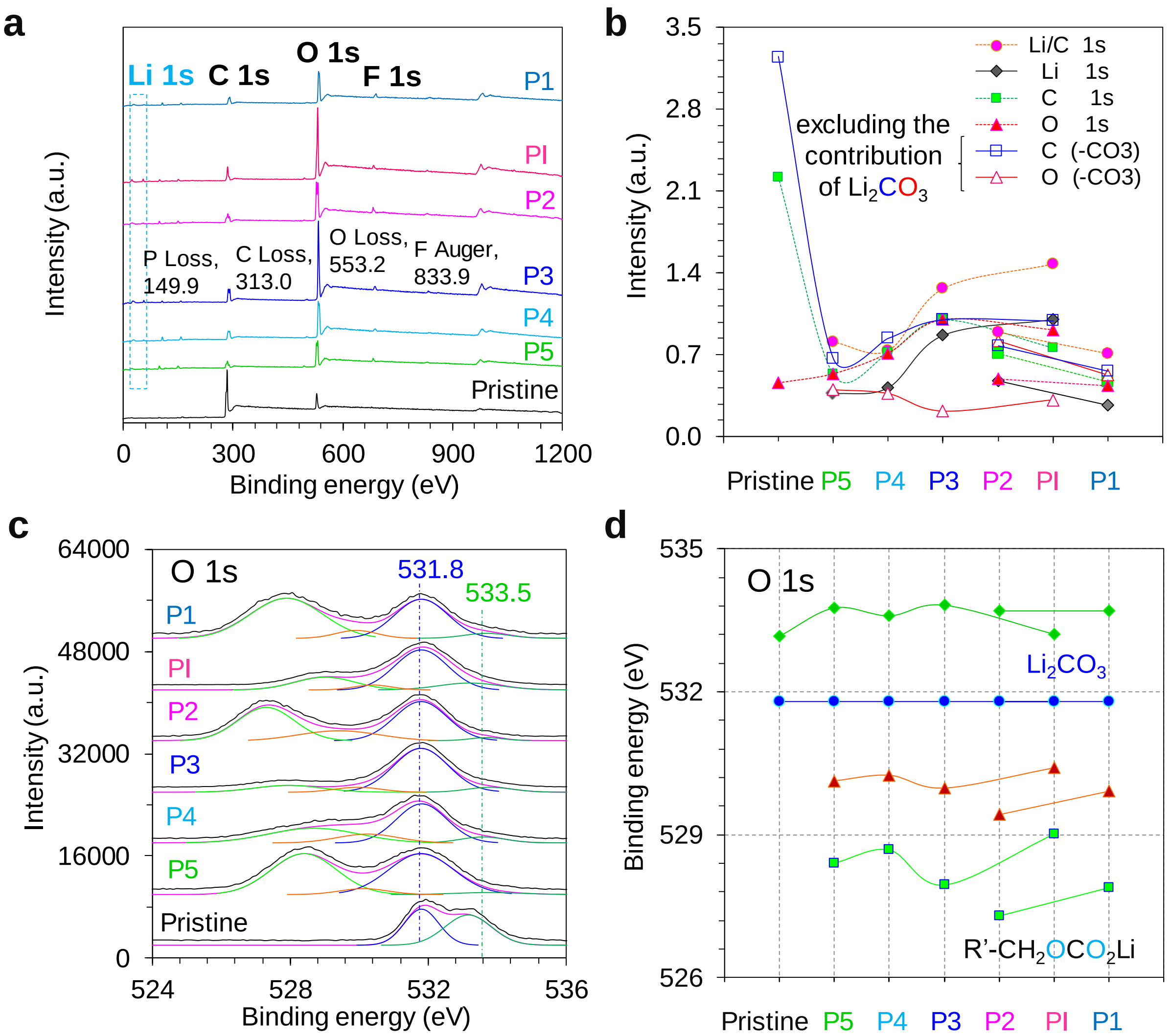

Supplementary Fig. 21 | Chemical composition identification of the bilayered LiC$_x$ phases during the Li-intercalation process. a, Full XPS spectra for pristine bilayer graphene and its lithiated phases during the Li-intercalation process. **b**, Evolution of the Li, C, and O contents and the Li/C ratios. Li species came from both of the SEI film (e.g., Li_2CO_3 and Li-contained organic molecules like R'-CH_2OCO_2Li[4]) and the interlayer. **c**,**d**, O 1s XPS spectra and the evolution of binding energies of the O 1s species in the SEI film. Similar to the C 1s and Li 1s cases, a regular trend also held for the O 1s spectra (**b**–**d**)[4,44]. Such phenomena (in particular the changes in the surface compositions) should reflect the outer surface adsorption capabilities of the lithiated bilayer-graphene phases with specific stacking configurations and Li densities (refer to the *in-situ* Raman study in Supplementary Fig. 12 and the composite EELS mapping images in Supplementary Fig. 15). Besides, as discussed at Supplementary Figs 9 and 16, the practical P2 sample should be a mixture of PI and P3 (or more). Accordingly, the P1 product is also supposed to be a mixture due to the local segregation and aggregation of Li atoms (known as the defect-induced Li plating behavior[38]), as reflected by its EELS mapping images (Supplementary Fig. 15a,c) and its similar XPS profiles to those of P2.

Supplementary Table

Supplementary Table 1. Simulated diffraction-spot intensity for bilayer graphene with different configurations and varied Li concentrations. The intensity values are shown by their logarithms (refer to Supplementary Note 2).

Simulated configuration		A-B stacking				A-A stacking				Graphene
		C_6LiC_6	$C_{12}LiC_{12}$	$C_{14}LiC_{14}$	C_6C_6	C_6LiC_6	$C_{12}LiC_{12}$	$C_{14}LiC_{14}$	C_6C_6	C_6
Logarithm relationship	Center	13.8155	13.8155	13.8155	13.8155	13.8155	13.8155	13.8155	13.8155	13.8155
	{0110}	5.0463	5.1242	5.1322	5.2102	6.2375	6.2907	6.3101	6.3436	5.5595
	{1210}	6.0739	6.0507	6.0455	6.0240	6.0800	6.0575	6.0498	6.0326	5.2896
Intensity ratio	$I_{\{0110\}}/I_{\{1210\}}$	0.358	0.396	0.401	0.443	1.171	1.263	1.297	1.365	1.310

Supplementary Notes

Supplementary Note 1. For the *in-situ* Raman spectra results (Supplementary Fig. 12)

In-situ Raman spectra characterization was carried out to check the stability of the defective sites on the lithiated graphene and to trace the Li intercalation/deintercalation process performed in the CV modes. Besides of three characteristic peaks assigned to the bilayer graphenic carbon (i.e., *D* band at 1352 cm^{-1}, *G* band at 1582 cm^{-1}, and *2G* band at 2702 cm^{-1}, Fig. 1e)[13,32], multiple peaks from the polymer electrolyte were observed from the evolutionary spectra. They remained stable enough in both of their shift positions and intensities through a whole cyclic process, indicating the steady state of the electrolyte and thus the SEI film during the operando measurements. The inconspicuous variation trend for the *D* bands further suggested that there were no distinct defects generating during the sluggish CV process. This result, as confirmed by the similar phenomena observed at both 0.1 and 0.02 mV s^{-1}, was different from either the previous *ex-situ* study[31] or our *ex-situ* Raman observation by using the oxidized samples (see the two reasons given at Supplementary Fig. 13c). Originating from a second-order double-resonant Raman scattering mechanism, the 2*D* peak also showed no obvious response to the Li intercalation (or the doping of holes and electrons) in its position and intensity (irrespective of the peak shape here due to the very high signal-to-noise ratio)[18,28−33]. Different circumstances took place on the *G* band induced by doubly discrete E_{2g} mode at the Brillouin zone center[13,32]. In particular, during the Li intercalation process (from 0.25 to 0.001 V), the intensity of the *G* band with a blue shift (up to 10 cm^{-1}) gradually decreased and began to vanish from 0.09 V (corresponding to the P3 phase). The intensity phenomenon can be attributed to the interference of the E_{2g2} mode with Raman active continuum, when the chemical shift has ever been considered to be induced by electron and hole doping[2,32,33] (as well as the varying train[28,45]), well suggesting the imbedding of Li atoms at the interlayer which can greatly influence the vibrational mode associated with their surrounding C atoms. The fundamental change after 0.09 V should also suggest the stacking configuration transformation at the P2 stage from A*R*A to A*R*B, in good line with the following XRD (Supplementary Fig. 16) and XPS (Fig. 4a,c and Supplementary Fig. 21c) results (refer to the quite different XRD and XPS data for P2 from those for P3 and P4). Meanwhile, the ever-increasing I_{2D}/I_G ratio, together with the blueshift of the *G* band[32], could imply the increasingly evident monolayer-like feature for the gradually lithiated graphene sample[11,15], in line with the *ex-situ* SAED phenomena (Fig. 3a). The rising I_D/I_G ratio[31] (particular from 0.1 V), together with the blueshift of *G* band[32,46], may indicate the ever-reduced size of the Li-intercalated graphene domains (refer to various LiC_x units in Supplementary Fig. 9b). Besides, an undefined and extrinsic *X* band at ca. 1856 cm^{-1} was noticed to emerge and fade away periodically under the necessary laser irradiation (Supplementary Fig. 12c−e), which should be bound up with both the electrolyte and the increasing Li population in light of its ever-changing intensity but stable chemical shift (similar to those typical bands for the electrolyte). To be specific, in view of the charge distribution in the LiC_x units (e.g., ca. +0.9|e| for Li in the C_6-Li constitutional unit)[3,40,41], Li-contained organic molecules (e.g., the R'-CH_2OCO_2Li presence in the SEI) may tend to directionally adsorb on the

outer surface of the graphene sheet with electronegativity[47], which eventually led to the occurrence and transformation of this new Raman band. The electrical nature of each LiC_x unit (with specific stacking configuration) may also influence its adsorbing capability for these Li-bearing organic adsorbents (refer to the EELS and O 1s XPS results in Supplementary Figs 15 and 21), which can protect the Li species from the direct irradiation of the laser beam (refer to the frustrated Raman acquisition at the slower 0.02 mV s^{-1} in Supplementary Fig. 12d) or also the electron beam (refer to the HR-TEM results in Supplementary Fig. 14).

Supplementary Note 2. For the simulated diffraction-spot intensity (Supplementary Table 1)

On the one hand, the $I_{\{0110\}}/I_{\{1210\}}$ ratio for each A-A stacking sample is always larger than 1, when the value is lower than 0.5 for the A-B ones. On the other hand, the Li intercalation into the interlayer tends to enhance the intensity of the {1210} diffraction spots and weaken that of the {0110} spots, yielding a reduced $I_{\{0110\}}/I_{\{1210\}}$ ratio for either of the configurations. This distinct difference from the experimental phenomena (Fig. 3a(i,iii)) further reflects the electron beam-irradiation effect fully discussed in Supplementary Figs 17−19.

Supplementary References

1. Raccichini, R., Varzi, A., Wei, D. & Passerini, S. Critical insight into the relentless progression toward graphene and graphene-containing materials for lithium-ion battery anodes. *Adv. Mater.* **29,** 1603421 (2017).
2. Pollak, E. *et al*. The interaction of Li^+ with single-layer and few-layer graphene. *Nano Lett.* **10,** 3386–3388 (2010).
3. Yao, F. *et al*. Diffusion mechanism of lithium ion through basal plane of layered graphene. *J. Am. Chem. Soc.* **134,** 8646–8654 (2012).
4. Radhakrishnan, G., Cardema, J. D., Adams, P. M., Kim, H. I. & Foran, B. Fabrication and electrochemical characterization of single and multi-layer graphene anodes for lithium-ion batteries. *J. Electrochem. Soc.* **159,** A752–A761 (2012).
5. Gabriel, A., Chatillon, C. & Ansara, I. Thermochemical and phase diagram analysis of the Ni-C, Co-C and Co-Ni-C systems. *High Temp. Sci.* **25,** 17–54 (1988).
6. Al-Shurman, K. M. & Naseem, H. CVD graphene growth mechanism on nickel thin films, *The Proceedings of the 2014 COMSOL Conference*, Boston, MA, p. **7** (2014).
7. Yu, Q. K. *et al*. Graphene segregated on Ni surfaces and transferred to insulators. *Appl. Phys. Lett.* **93,** 113103 (2008).
8. Ito, Y. *et al*. High-quality three-dimensional nanoporous graphene. *Angew. Chem. Int. Ed.* **53,** 4822–4826 (2014).
9. Wu, T. R. *et al*. Fast growth of inch-sized single-crystalline graphene from a controlled single nucleus on Cu–Ni alloys. *Nat. Mater.* **15,** 43–47 (2016).
10. Lee, H. C. *et al*. Review of the synthesis, transfer, characterization and growth mechanisms of single and multilayer graphene. *RSC Adv.* **7,** 15644–15693 (2017).
11. Meyer, J. C. *et al*. The structure of suspended graphene sheets. *Nature* **446,** 60–63 (2007).
12. Meyer, J. C. *et al.* On the roughness of single- and bi-layer graphene membranes. *Solid State Commun.* **143,** 101–109 (2007).
13. Kim, K. *et al.* Raman spectroscopy study of rotated double-layer graphene: Misorientation-angle dependence of electronic structure. *Phys. Rev. Lett.* **108,** 246103 (2012).
14. Kim, K. *et al.* Multiply folded graphene. *Phys. Rev. B* **83,** 245433 (2011).
15. Hernandez, Y. *et al*. High-yield production of graphene by liquid-phase exfoliation of graphite. *Nat. Nanotech.* **3,** 563–568 (2008).

16. Shi, G. *et al*. Advancement in liquid exfoliation of graphite through simultaneously oxidizing and ultrasonicating. *J. Mater. Chem. A* **2,** 20382−20392 (2014).
17. Horiuchi, S. *et al*. Carbon nanofilm with a new structure and property. *Jpn. J. Appl. Phys.* **42,** L1073–L1076 (2003).
18. Ferrari, A. C. *et al*. Raman spectrum of graphene and graphene layers. *Phys. Rev. Lett.* **97,** 187401 (2006).
19. Pyun, S. -I. & Ryu, Y. Lithium transport through graphite electrodes that contain two stage phases. *J. Power Sources* **70,** 34–39 (1998).
20. Kashani, H. *et al*. Bicontinuous nanotubular graphene–polypyrrole hybrid for high performance flexible supercapacitors. *Nano Energy* **19,** 391–400 (2016).
21. Levi, M. D., Levi, E. A. & Aurbach, D. The mechanism of lithium intercalation in graphite film electrodes in aprotic media. *J. Electroanal. Chem.* **421,** 79–97 (1997).
22. Sacci, R. L., Gill, L. W., Hagaman, E. W. & Dudney, N. J. Operando NMR and XRD study of chemically synthesized LiC_x oxidation in a dry room environment, *J. Power Sources* **287,** 253–260 (2015).
23. Umeda, M. *et al.* Electrochemical impedance study of Li-ion insertion into mesocarbon microbead single particle electrode: Part I. Graphitized carbon. *Electrochim. Acta* **47,** 885–890 (2001).
24. Levi, M. D., Wang, C., Gnanaraj, J. S. & Aurbach, D. Electrochemical behavior of graphite anode at elevated temperatures in organic carbonate solutions. *J. Power Sources* **119–121,** 538–542 (2003).
25. Shim, J. & Striebel, K. A. The dependence of natural graphite anode performance on electrode density. *J. Power Sources* **130,** 247–253 (2004).
26. NuLi, Y., Yang, J. & Jiang, Z. Y. Intercalation of lithium ions into bulk and powder highly oriented pyrolytic graphite. *J. Phys. Chem. Solids* **67,** 882–886 (2006).
27. Rui, X. H., Yesibolati, N., Li, S. R., Yuan, C. C. & Chen, C. H. Determination of the chemical diffusion coefficient of Li^+ in intercalation-type $Li_3V_2(PO_4)_3$ anode material. *Solid State Ionics* **187,** 58–63 (2011).
28. Ferralis, N. Probing mechanical properties of graphene with Raman spectroscopy. *J. Mater. Sci.* **45,** 5135–5149 (2010).

29. Lee, J. -U. *et al.* Polarization dependence of double resonant Raman scattering band in bilayer graphene. *Carbon* **72,** 257–263 (2014).

30. Huang, C. -W. *et al.* Probing 2D sub-bands of bi-layer graphene, *RSC Adv.* **4,** 51067–51071 (2014).

31. Sethuraman, V. A., Hardwick, L. J., Srinivasan, V. & Kostecki, R. Surface structural disordering in graphite upon lithium intercalation/deintercalation. *J. Power Sources* **195,** 3655−3660 (2010).

32. Das, A., Chakraborty, B. & Sood, A. K. Raman spectroscopy of graphene on different substrates and influence of defects. *Bull. Mater. Sci.* **31,** 579–984 (2008).

33. Das, A. *et al*. Monitoring dopants by Raman scattering in an electrochemically top-gated graphene transistor. *Nat. Nanotech.* **3,** 210−215 (2008).

34. Sasaki, K. -I., Tokura, Y. & Sogawa, T. The origin of Raman *D* band: bonding and antibonding orbitals in graphene. *Crystals* **3,** 120–140 (2013).

35. Yao, W., *et al.* Quasicrystalline 30° twisted bilayer graphene as an incommensurate superlattice with strong interlayer coupling. *Proc. Natl. Acad. Sci.* **115,** 6928–6933 (2018).

36. Ahn, S. J., *et al*. Dirac electrons in a dodecagonal graphene quasicrystal. *Science* **361,** 782–786 (2018).

37. Chattopadhyay, S. *et al*. In situ X-ray study of the solid electrolyte interphase (SEI) formation on graphene as a model Li-ion battery anode. *Chem. Mater.* **24,** 3038–3043 (2012).

38. Mukherjee, R. *et al*. Defect-induced plating of lithium metal within porous graphene networks. *Nat. Commun.* **5,** 3710 (2014).

39. Wang, Z. H., Selbach, S. M. & Grande, T. Van der Waals density functional study of the energetics of alkali metal intercalation in graphite. *RSC Adv.* **4,** 4069–4079 (2014).

40. Zhou, L. J., Hou, Z. F. & Wu, L. M. First-principles study of lithium adsorption and diffusion on graphene with point defects. *J. Phys. Chem. C* **116,** 21780–21787 (2012).

41. Mordkovich, V. Z. Synthesis and XPS investigation of superdense lithium-graphite intercalation compound, LiC_2. *Synthetic Met.* **80,** 243–247 (1996).

42. Dahn, J. R. Phase diagram of Li_xC_6. *Phys. Rev. B* **44,** 9170−9177 (1991).

43. Takami, N., Satoh, A., Hara, M. & Ohsaki, T. Structural and kinetic characterization of lithium intercalation into carbon anodes for secondary lithium batteries. *J. Electrochem. Soc.* **142,** 371–379 (1995).

44. Hori, H. *et al*. Analysis of hard carbon for lithium-ion batteries by hard X-ray photoelectron spectroscopy. *J. Power Sources* **242,** 844–847 (2013).

45. Oliveira Jr., M. H. *et al.* Formation of high-quality quasi-free-standing bilayer graphene on SiC (0001) by oxygen intercalation upon annealing in air. *Carbon* **52**, 83−89 (2013).

46. Tuinstra, F. & Koenig, J. L. Raman spectrum of graphite. *J. Chem. Phys.* **53,** 1126–1130 (1970).

47. Momose, H. *et al*. X-ray photoelectron spectroscopy analyses of lithium intercalation and alloying reactions on graphite electrodes. *J. Power Sources* **68,** 208–211 (1997).
